# The rise and fall of cooperation through reputation and group polarization

**DOI:** 10.1038/s41467-019-08727-8

**Published:** 2019-02-15

**Authors:** Jörg Gross, Carsten K. W. De Dreu

**Affiliations:** 1 Department of Psychology, Leiden University, P.O. Box 9555, 2300 RB Leiden, The Netherlands; 2 Center for Research in Experimental Economics and Political Decision Making (CREED), University of Amsterdam, P.O. Box 1551, 1001 NB Amsterdam, The Netherlands

## Abstract

Humans exhibit a remarkable capacity for cooperation among genetically unrelated individuals. Yet, human cooperation is neither universal, nor stable. Instead, cooperation is often bounded to members of particular groups, and such groups endogenously form or break apart. Cooperation networks are parochial and under constant reconfiguration. Here, we demonstrate how parochial cooperation networks endogenously emerge as a consequence of simple reputation heuristics people may use when deciding to cooperate or defect. These reputation heuristics, such as “a friend of a friend is a friend” and “the enemy of a friend is an enemy” further lead to the dynamic formation and fission of cooperative groups, accompanied by a dynamic rise and fall of cooperation among agents. The ability of humans to safeguard kin-independent cooperation through gossip and reputation may be, accordingly, closely interlinked with the formation of group-bounded cooperation networks that are under constant reconfiguration, ultimately preventing global and stable cooperation.

## Abstract (web summary)

Group membership can inform individuals’ decisions on whether to cooperate. Here, the authors show how cooperative groups themselves can emerge and change due to use of reputation heuristics (such as “the enemy of a friend is an enemy”), and how this destabilizes cooperation over time.

## Introduction

Compared to many other social animals, humans cooperate in networks of genetically unrelated individuals^1,2^, possibly because humans are uniquely capable to observe the actions of others^3^, track their reputation^4–7^, and exchange information on the trustworthiness of strangers through gossip^8–11^. Yet, cooperation among humans is neither universal nor stable. Throughout history, humans organized themselves into social groups characterized by high in-group cooperation and out-group defection^12–15^. Furthermore, cooperation within and between groups fluctuates and cooperation networks constantly change, reconfigure themselves^16–18^, or completely vanish^18,19^. Indeed, hunter gatherer societies sometimes fight, cooperate, or merge to larger groups that then break up again^18^. Likewise, throughout history, alliances and coalitions within and between nation states formed, fell apart, and re-emerged again^20,21^.

Why cooperative groups and networks of unrelated individuals form, break-up, and reconfigure themselves, can be explained well on the basis of human tendencies to rely on reputation and indirect reciprocity mechanisms^4,5,22–25^. Likewise, reputation and indirect reciprocity based on past experience or friendship can also explain why human cooperation is in-group bounded and hardly extends to members of out-groups^12,22,26–29^. To date, however, these two lines of discovery emerged in relative isolation. Moreover, past work on reputation and indirect reciprocity assumed some form of fixed group structure based on genetic relatedness or affiliation cues (“green beards”) to explain when and why both group fission-and-fusion and parochial cooperation can emerge^14,27,28,30–32^.

Here, we report simulations in which agents have private information on the cooperativeness of other interaction partners, exchange information on others (viz. gossip) and use such reputation information heuristically when deciding to cooperate with others. We find that without assuming relatedness or explicitly modelling group affiliation, a set of intuitively plausible adaptations in the reputation heuristics can lead to (i) the dynamic emergence of group structures, that are (ii) under constant reconfiguration and (iii) marked by in-group bounded, “parochial” cooperation. Combined, our findings suggest that reputation heuristics can explain both the emergence of parochial group structures and the dynamic rise and fall of groups and cooperation networks among unrelated individuals.

## Results

### Model

Point of departure in our analysis is a population of agents (e.g., individuals or groups) that randomly meet and interact with each other. They have the option to cooperate or defect. When two agents cooperate, they strengthen their relationship by *r*. However, if the opponent decides to defect, the agent decreases its relationship with this agent by *r*. Before deciding to cooperate or defect, they both consult other agents in the population about their relationship with, and hence opinion about, the other agent. They do not trust this opinion blindly, but weigh it by their own relationship with the agent that they receive an opinion from. This leads to four reputation heuristics first described by Heider^33^, that determine the likelihood that an agent A will cooperate with another agent B. An example may illustrate that; Agent A has a positive relationship with agent C and C has a positive opinion about B. This increases A’s likelihood to cooperate with B, since “a friend of a friend is a friend”. A also has a positive relationship with agent D who has a negative opinion about B. This will decrease A’s likelihood to cooperate with B, since “an enemy of a friend is an enemy”. Further, A has a negative relationship with E who is positive about B. This will further decrease A’s likelihood to cooperate with B, since “a friend of an enemy is an enemy”. And lastly, A has a negative relationship with agent F who is negative about B, which will increase A’s likelihood to cooperate with B, since “an enemy of an enemy is a friend”.

While these four reputation heuristics exhaust all possible configurations, they are variably applied. Sometimes, cooperation emerges on the basis of the last two “enemy” heuristics. During the cold war, for example, the US allied with the Afghan Mujahedeen to fight their common enemy, the Soviets. However, such “enemy” heuristics require that agents take the opinion of those with whom they have a negative relationship into account. Agents may not do this, because they are simply not interested in the opinion of agents they have a negative relationship with, they distrust and discount information from such agents, or such agents are not forthcoming with reputation information. In all these cases, decisions to cooperate have to be based on the first two “friendship” heuristics only. Accordingly, we introduce two types of agents—Heider agents and friend-focused agents—in a population of size *n*. Whereas Heider agents take opinions of both friends and enemies into account, hence rely on all four reputation heuristics, friend-focused agents only consult friends in their decision to cooperate (“a friend of a friend is a friend” and “an enemy of a friend is an enemy”). Reputation based on Heider-rules can be represented in an *m* × *n* reputation matrix in which the column vector **n**_*y*_ represents the opinions agents have about an agent *y*, the row vector **m**_*x*_ represents the relationships that an agent *x* has with all other agents, and **m**_*x*_ × **n**_*y*_ is the aggregated weighted opinion of an agent *x* towards an agent *y*. This aggregated weighted opinion determines the likelihood that agent *x* cooperates or defects when meeting agent *y*. For friend-focused agents, **m**_*x*_ is replaced by ${\mathbf{m}}_x^\prime$ where ${\mathbf{m}}_x^\prime$ = max{0, m_*x*_}.

### Network polarization

**Fig. 1:**
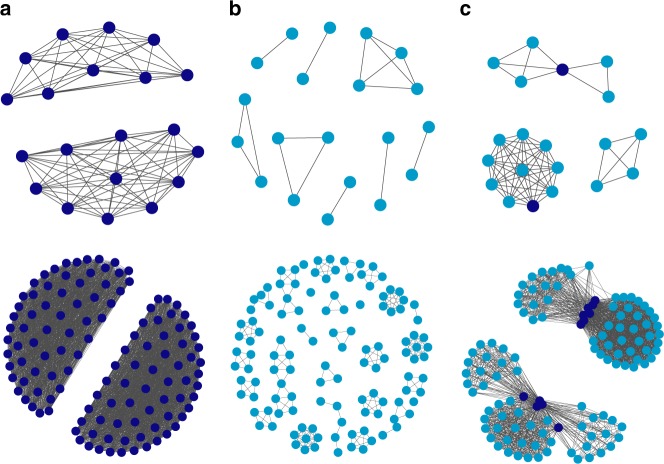
Polarization through reputation. Emerging community networks in small (*n* = 20, upper panel) and large (*n* = 120, lower panel) populations of **a** Heider agents, **b** friend-focused agents, and **c** a majority of friend-focused agents (light blue dots) together with a minority (10%) of Heider agents (dark blue dots). Links between agents represent mutually positive relationships between two agents and hence a high likelihood to interact cooperatively

Through multiple encounters and dynamic relationship updating based on these rules, a population of Heider agents enters a balanced state of one large group (with probability *p* = 0.07, based on simulations with group-sizes between 10 and 120) or, more likely (with *p* = 0.93), two groups marked by high in-group cooperation and out-group defection (Fig. 1a). Under the same parameters, a population of friend-focused agents build smaller, more scattered communities, marked by high cooperation within these communities but no cooperation across communities (Fig. 1b). We refer to this transition from many small communities to a few large communities as polarization. A population of Heider agents with two opposing groups is hence maximally polarized. But what happens in mixed populations of Heider and friend-focused agents? As exemplified in Fig. 1c, already a minority of Heider agents can lead to a great increase in group-size, and hence a more polarized network state.

**Fig. 2:**
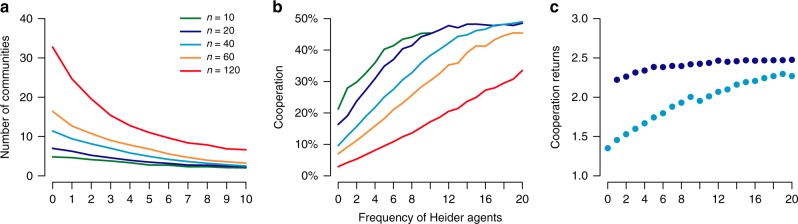
Heider agents increase cooperation, group welfare, and group polarization. **a** The average number of communities decreases (measured by the Louvain method for community detection^34^). Hence, the population becomes more polarized, as the number of Heider agents increases, across different population sizes (green line: *n* = 10, dark blue line: *n* = 20, light blue line: *n* = 40, yellow line: *n* = 60, red line: *n* = 120). **b** Meanwhile, cooperation rates increase with increasing numbers of Heider agents, and **c** friend-focused agents (light blue dots) benefit from Heider agents (dark blue dots), as their average welfare increases

With every additional Heider agent in the population, and across varying population sizes, the number of communities (i.e., groups of agents that are densely interconnected within, but not between groups, as measured by the Louvain method for community detection^34^) exponentially declines by a factor of *τ* = 4.9 (exponential decay regression, Fig. 2a). Alongside a more polarized state of the cooperation network, a small number of Heider agents increases cooperation due to larger and more densely interconnected communities. More specifically, with every additional Heider agent, population-level cooperation increases by a factor of 1 − *τ* between 5.5 (*n* = 10) and 83.7 (*n* = 120; Fig. 2b). Especially friend-focused agents benefit from Heider agents, as their cooperation-rates increase (Fig. 2c). In short, simple reputation rules based on gossip and reputation memory can lead to closely interconnected groups that have clear group boundaries marked by high in-group cooperation and out-group defection. Already a few Heider agents can starkly influence group formation and group size.

### Evolutionary dynamics

To see whether reputation-based decisions to cooperate or defect influence the agent’s relative success in the population and are evolutionary stable against pure defection, agents engaged in a Prisoner’s Dilemma. Playing C costs *c* and gives the other agent benefit *b*, where *b* > *c*, while playing D is costless and does not benefit the other agent, *b* = *c* = 0. After repeated interactions, payoffs influenced the likelihood that an agent’s strategy would spread in the population or die out. Specifically, after *i* periods, one agent is randomly selected to adapt its strategy. With probability *u*, the agent adopts a strategy at random (random mutation). With probability *1* − *u* the agent adopts a strategy of another agent in the population based on the relative success of this agent (which mimics genetic evolution or social learning). Further, we introduced a third type of agent, the always-defect type (or simply “defectors”/“free-riders”), that attempts to take advantage of other agents by always playing the selfish option D.

**Fig. 3:**
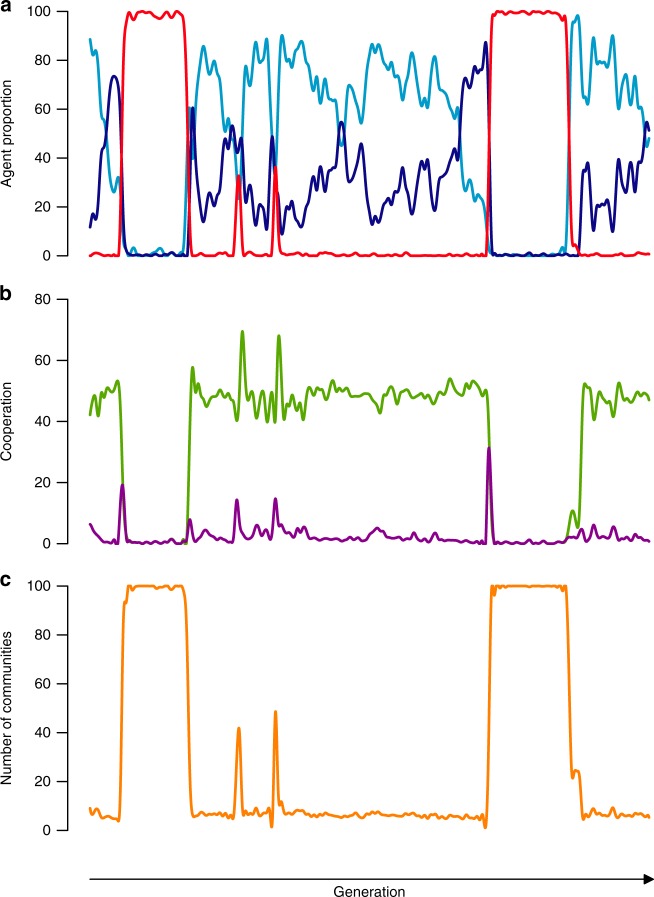
Fission–fusion dynamic of reputation-based cooperation. As the number of Heider agents (dark blue line) in a population of friend-focused agents (light blue line) increases, the risk of invasion by free-riders (red line) increases (**a**). A population of free-riders in turn gets invaded by friend-focused agents. Following this dynamic, cooperation (green line) and successful exploitation (magenta line) fluctuates (**b**) and community structures emerge and dissolve as a function of the population dynamic (**c**)—based on *n* = 100, 4 × 10^5^ iterations, *i* = 10, *c* = 1, *b* = 4, *r* = 0.3

Figure 3 shows the observed evolutionary dynamic across time. In high cooperation periods, the population consists of a majority of friend-focused and a minority of Heider agents (Fig. 3a). However, Heider agents eventually spread, take over, and polarize the population. At this stage, the population becomes vulnerable to invasion by defectors. This follows from the fact that Heider agents are more likely to cooperate with isolated agents, because of shared negative connections to other agents (the “enemy of my enemy is my friend” principle, see also Supplementary Note 2). While in combination with friend-focused agents, this characteristic helps to make connections with other groups, Heider agents are unable to systematically isolate defectors. Thus, Heider’s four reputation principles and the concept of psychological transitivity, are highly exploitable by free-riders. As a result, cooperation declines and because the population transitions to a state of defection, the group structures dissolve. In this state, friend-focused agents can emerge again and build small isolated communities that strictly cooperate with their in-group. After spreading, single Heider agents appear again and increase both cooperation and community-size. In short, we observe a dynamic rise and decline of cooperation (Fig. 3b), accompanied by cycles of group-formation and group-disintegration (Fig. 3c, Supplementary Figure 1).

**Fig. 4:**
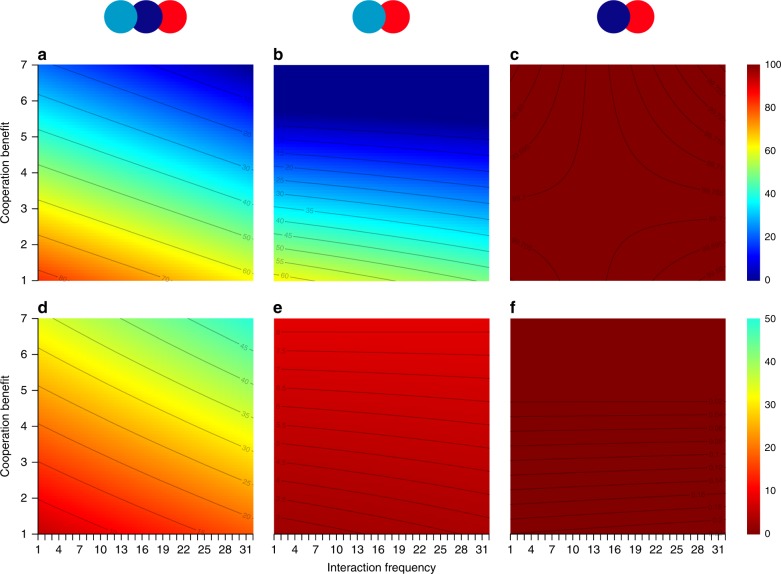
Cooperation and free-riding across the parameters space. The proportion of free-riders (upper row) decreases with higher interaction frequency *i* and return on cooperation *b* with all three-strategies (**a**) and friend-focused vs. free-rider strategies (**b**), but free-riding dominates when Heider agents compete against free-riders (**c**). Cooperation (in percentage of interactions—lower row) only reaches significant levels with all three strategies present (**d**) and remains low in friend-focused vs. free-rider (**e**) and Heider vs. free-rider populations (**f**)

The speed of this evolutionary dynamic and survivability of cooperation strategies depends on the benefit of cooperation and the interaction frequency. With higher interaction frequency and return of cooperation, the relative proportion of defectors in the population decreases (Fig. 4a) and mutual cooperation increases (Fig. 4d; see also Supplementary Note 1).

### Pairwise invasions

We verified to which extent these dynamics depend on the interaction of friend-focused and Heider agents by repeating the simulations with one single agent type (either friend-focused or Heider agents) performing against free-riders (see also Supplementary Note 2 and 4). We find that without friend-focused agents, Heider agents alone do not survive against free-riders (Fig. 4c). Friend-focused agents without Heider agents, on the other hand, survive against free-riders (Fig. 4b), but only build small communities that result in very low population-wide cooperation (Fig. 4e). Hence, both friend-focused and Heider agents are needed to achieve periods of high, albeit unstable, cooperation.

**Fig. 5:**
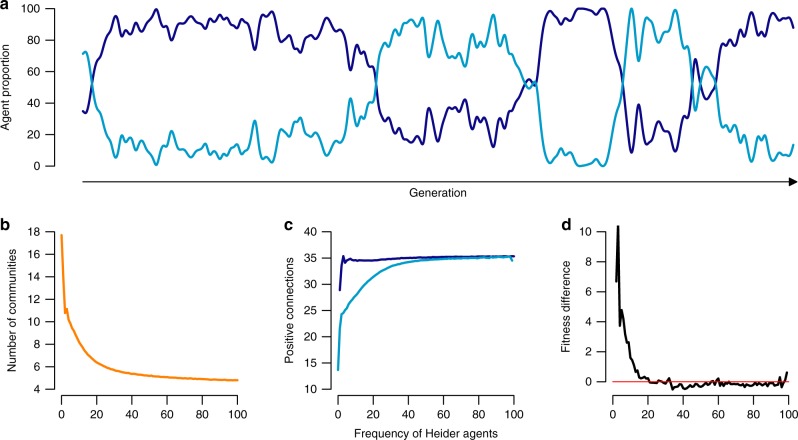
Co-existence of Heider and friend-focused agents. Heider and friend-focused agents can co-exist and a population of Heider and friend-focused agents constantly transitions from one majority state to the other (**a**)—based on *n* = 100, 4 × 10^5^ iterations, *i* = 10, *c* = 1, *b* = 4, *r* = 0.3. As observed in the simulations without mutations, the increase of Heider agents decreases the number of communities in the population (**b**). The ability of Heider agents (dark blue line) to make connections to isolated agents outside of their friendship-network initially leads to more (positive) outgoing connections compared to friend-focused agents (light blue line) (**c**). The difference in connectedness is accompanied by a fitness advantage over friend-focused agents that diminishes, once the group structure is established (positive numbers indicate higher fitness for Heider agents) (**d**)—based on *n* = 100, averaged over 5 × 10^7^ iterations, *i* = 10, *c* = 1, *b* = 4, *r* = 0.3

As we can see in Fig. 3a, Heider agents do not strictly dominate friend-focused agents, leading to periods of co-existence of these two types. In simulations without free-riders, we can examine this dynamic more closely (Fig. 5a). Replicating the findings without selection pressure (Fig. 2), an increase of Heider agents is accompanied by a decrease in the number of communities (*τ* = 13.2, exponential decay regression)—the polarization effect of the full Heider heuristics. Importantly, the ability of Heider agents to establish positive connections to agents outside of the friendship network (“the enemy of my enemy is my friend”) leads to an initial advantage over friend-focused agents. They form more positive outgoing connections (Fig. 5c) and have higher relative fitness, initially (Fig. 5d). As Heider agents spread in the population, this gap between Heider agents and friend focused agents disappears. Friend-focused agents take advantage of the more polarized network structure that is established by Heider agents. Eventually, friend-focused agents have the same fitness as Heider agents (Fig. 5d). In this state, the population can make a neutral drift to friend-focused agents again. The invasion-success of Heider agents in a population of friend-focused agents depends on the benefit of cooperation and the interaction frequency. Only with moderate to high interaction frequency, Heider agents have enough time to polarize the network and their initial advantage over friend-focused agents is higher with higher returns of cooperation (see also Supplementary Note 2).

### Memory constraints

Results thus far were constrained by assuming that agents can consult all other agents in the population and were able to take their opinion into account. Realistically, however, the ability to process information about others is constrained by and depends on cognitive abilities like memory capacity. Such cognitive abilities considerably changed throughout natural evolution^35,36^ and the access to and exchange of opinions may have changed throughout human history as a function of the ability to write and read, the flow of information through logistic systems like mass media, and innovations in information technology like the internet. We therefore modelled information constraints by allowing agents to only store opinions of a restricted number of *k* agents, with whom the agent has the most extreme relationships. Information constraint can be either considered a limitation on cognitive capacity of agents (i.e., memory) or limited information flow based on cultural development.

**Fig. 6:**
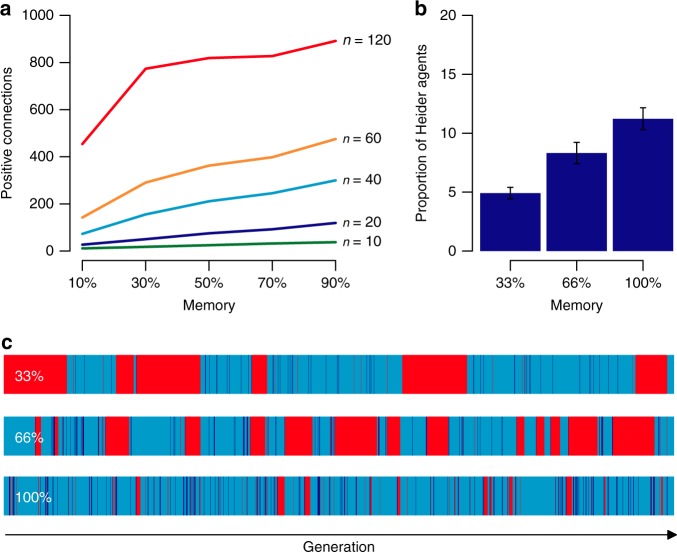
Reputation and memory. With increased memory capacity (percentage of memorized relationships), reputation sensitive agents establish more cooperative connections (**a**). When under selection pressure, an increase in memory also increases the relative proportion of Heider agents in the population (**b**), which leads to more rapid cycles (**c**) between a population that consists of a majority of friend-focused agents (light blue columns), Heider agents (dark blue columns) and free-riders (red columns)—based on *n* = 100, 10^5^ iterations, *i* = 10, *c* = 1, *b* = 4, *r* = 0.3. Error bars indicate the standard error of the mean

We find that with larger memory, cooperative network relationships sharply increase among reputation sensitive agents (Fig. 6a, *β* = 5.4). Further, in competition with free-riders, the relative proportion of Heider agents among reputation-sensitive agents increases by *β* = 0.1 percentage points per memory bit (Fig. 6b). Larger memory, hence, increases global cooperation (*β* = 0.3 percentage points per memory bit), but also leads to faster defection-cooperation cycles and more rapid fission-fusion group dynamics (Fig. 6c). We observe 6, 19, and 24 defection–community building–polarization cycles per 1000 generations for low, medium, and high memory and information transmission, respectively (see also Supplementary Note 3). Hence, higher transmission capacity of reputation information increases the speed and interconnectedness of group-bounded cooperation at the cost of faster reconfigurations and fission–fusion dynamics.

## Discussion

Others before us noted that the unique capability of complex symbolic communication paired with large episodic memory, conceivably driven by the reorganization of the prefrontal cortex throughout primate evolution^37,38^, may have allowed humans to cooperate on a large scale, independent of genetic relatedness^23,39^. Our results shed light on how such cooperation can emerge based on memory, gossip, and simple engagement rules. Heider’s reputation heuristics align well with real-world social structures, including interpersonal relationships^40^ and international alliances and coalitions^41^. Our findings also resonate with results from behavioral experiments on the role of reputation, group formation, and memory in cooperation^17,42–46^. In particular, it has been shown that more information on the past actions of other players (i.e., memory) influences network formation and leads to a higher frequency of cooperation^43^. Further, information exchange on past actions can increase cooperation^47,48^ (see however ref. ^42^), and participants readily share gossip on the cooperativeness of interaction partners, which subsequently increases cooperation^10^. Reminiscent of the “a friend of my friend is my friend” heuristic, experiments have shown that humans integrate reputation information about others through gossip^10,49^, that humans condition their decisions to cooperate on gossip received from others, with cooperation being increased (withheld) when gossip suggests the partner could (not) be trusted^10,49,50^. This in turn mediates the formation of social networks and communities^51^. Relatedly, work on extended intergroup contact shows that knowledge of a friend’s positive contact with an out-group member leads people to develop more positive attitudes towards that out-group themselves^52–54^, suggesting that intergroup relations can change as a function of indirect reciprocity. The operation of the “a friend of my enemy is my enemy” heuristic is seen in work on vicarious retribution whereby an individual aggresses out-group members affiliated with an out-group member who harmed some in-group member other than the individual him or herself^55^.

In our simulations, Heider’s reputation heuristics, and adaptations therein, can also account for the dynamic increase and decline of cooperation within and between (groups of) individuals, the fission–fusion dynamics of groups of unrelated individuals and, perhaps even the rise and fall of civilizations as seen throughout human history^19^. Especially cooperation based on mutual enmity towards third parties (“the enemy of my enemy is my friend”) operates as a double-edged sword: It leads to larger and more interconnected groups, but to more polarized networks in which whole populations become vulnerable to defection. Friend-focused agents, in contrast, successfully shield themselves against defectors at the price of smaller friendship networks and low population-wide cooperation, revealing a trade-off between exclusively cooperating in small friendship-networks and attempting to cooperate with agents outside of the friendship-network at the risk of exploitation.

The oscillation between cooperation and defection is a recurrent theme in the evolution of cooperation that has also been observed in models based on tags (“green beard”)^27,32^, voluntary public goods participation^56^, direct reciprocity^57^, imitation^26^, pool-punishment^58,59^, spatial migration^60^, and anti-social punishment^61,62^ (see ref. ^63^ for a review). Going beyond clear group affiliation via tags (“green beards”)^27,64^, our results demonstrate that the reliance on reputation heuristics and gossip is sufficient to observe the emergence of dynamically changing group affiliations, group-bounded cooperation, as well as fluctuations in global cooperation among unrelated kin.

Previous work (e.g., refs. ^4,7,23,39^) has extensively investigated image-scoring rules that assign reputation based on the action of a “donor” and the reputation of a “receiver”, like: “help good people and refuse to help otherwise” (stern judging). This work led to eight rules that have been shown to stabilize cooperation through indirect reciprocity (“the leading eight”)^65,66^. Importantly, the “leading eight” rely on the ability to observe the actions of others in the population to a certain extent and apply a clearly defined social norm to assign reputation. In contrast, reputation based on Heider rules relies on private experiences of other agents weighted by own experiences with this agent. Agents value the opinion of another agent to the extent that they had good experiences with this agent. As such, Heider rules may be particularly important when observing actions is difficult but exchanging opinions is easy. As such, invoking Heider rules can help to understand the emergence of cooperative group-clusters even when social norms are not clearly defined and actions are based on personal affinity or enmity and gossip. Since private experience is noisy and also depends on chance (as in our model at initialization), arbitrary group boundaries emerge between agents that restrict the extent of population-wide cooperation even when the underlying decision-rules of agents are similar.

Beyond cooperation, the role of reputation and gossip in the emergence of groups may have important implication for attitude formation, how political opinions spread and polarize (e.g., ref. ^67^), or how selective information exchange shapes coalitions and rivalries. Our simulations finally suggest that human friendship-networks based on reputation and information transmission can considerably and quickly change with cultural development and modern technology. As long as cooperation is reputation-based, group structures can be volatile and cooperation among humans may not be, nor become, universal and stable.

## Methods

### Model

In our simulations, agents from a finite population of size *n* go through three discrete stages in each iteration: (1) Random matching. Every agent is randomly paired with another agent. (2) Action choice. Every agent chooses action {C, D}. (3) Relationship updating. Every agent updates their relationship with the paired agent.

The action-pair has consequences for the agents’ relationship. In case two agents *x* and *y* play (C, C), the relationship *s*_*xy*_ and *s*_*yx*_ increases by *r*. If the opponent plays D, the relationship decreases. Specifically, if the opponent *x* plays D, the relationship *s*_*yx*_ decreases by *r*. If the opponent *y* plays D, the relationship *s*_*xy*_ decreases by *r*. An agent *x* that defects, while the opponent *y* cooperates does not alter the relationship *s*_*xy*_, to avoid negatively correlated relationships between two agents across time (i.e., in round *t*, *x* is positive towards *y* and *y* is negative towards *x*, in round *t* + 1, *x* is negative towards *y* and *y* is positive towards *x* and so on).

Relationships can be represented in a quadratic *m* × *n* reputation matrix **S**. The diagonal represents the relationship every agent has with itself and is fixed to 1;

1 $$\begin{array}{*{20}{c}} {{\mathbf{S}}_{m,n} = \left( {\begin{array}{*{20}{c}} 1 & {s_{1,2}} & \cdots & {s_{1,n}} \\ {s_{2,1}} & 1 & \cdots & {s_{2,n}} \\ \vdots & \vdots & \ddots & \vdots \\ {s_{m,1}} & {s_{m,2}} & \cdots & 1 \end{array}} \right),\,{\mathrm{where}}\left\{ {s_{i,j} \in \,{\Bbb Q}{\mathrm{|}} - 1 \le s_{i,j} \le 1} \right\}\,{\mathrm{and}}\,m = n} \end{array}$$

Each row vector **m**_*x*_ (relationship vector) represents the relationship an agent *x* has with every other agent (and itself), while each column vector **n**_*x*_ (reputation vector) represents the opinion every agent has about agent *x* (i.e., their respective relationship with agent *x*).

For the main analyses, we define two reputation-sensitive agents that differ in how they determine when to play C or D.

### Heider agents

When paired with an agent *y*, a Heider agent *x* takes the reputation vector **n**_*y*_ and multiplies each element *i* (opinions about *y* of agent *i*) by their relationship with the respective agent *i*, leading to the relationship score rs = **m**_*x*_ × **n**_*y*_. If a population consists of Heider agents only, the relationship scores of the population are simply **S**^2^.

The relationship score is thus the weighted and aggregated product based on the four relationship heuristics, first outlined by Heider^33^: A friend of a friend is a friend (positive relationship *s*_*xi*_ and positive opinion *s*_*iy*_), an enemy of a friend is an enemy (positive relationship *s*_*xi*_ and negative opinion *s*_*iy*_), a friend of an enemy is an enemy (negative relationship *s*_*xi*_ and positive opinion *s*_*iy*_), an enemy of an enemy is a friend (negative relationship *s*_*xi*_ and negative opinion *s*_*iy*_).

### Friend-focused agents

Compared to Heider agents, a friend-focused agent *x* only takes opinions of friends into account. Friends are agents with whom the relationship *s*_*xi*_ > 0. The relationship-vector for a friend-focused agent is thus replaced with ${\mathbf{m}}_x^\prime$ where ${\mathbf{m}}_x^\prime$ = max{0, *m*_*x*_}. Accordingly, the relationship score rs = ${\mathbf{m}}_x^\prime$ × **n**_*y*_ is the weighted and aggregated product based on the opinions of friends. Friend-focused agents, hence, only act upon the two friend-heuristics: “a friend of a friend is a friend” and “an enemy of a friend is an enemy”.

The relationship score rs determines the probability to choose C based on the logistic decision function:

2 $$\begin{array}{*{20}{c}} {p\left( C \right) = \frac{1}{{1 + {\mathrm{exp}}( - {\mathrm{rs}}/0.2)}}\,{\mathrm{and}}\,1 - p\left( C \right) = p(D)} \end{array}$$

### Network polarization

In the simulations, agents repeatedly and randomly met, chose action {C, D}, and updated their relationship accordingly. Note that we specifically did not manipulate meeting probability based on relationship-score as in other models^68^, since cooperation and group structure become a function of meeting probability and cannot be disentangled anymore.

Supplementary Movies 1–3 demonstrate the emerging network structure in a population of *n* = 20 agents. The reputation matrix **S** is a 20 × 20 identity matrix at initialization and updated according to the rules described above. Supplementary Movie 1 shows the relationship network for 20 Heider agents, Supplementary Movie 2 shows the relationship network for 20 friend-focused agents, and Supplementary Movie 3 shows the relationship network for 16 friend-focused and a minority of 4 Heider agents.

For the results underlying Fig. 1, results were analyzed after 10^5^ iterations (i.e., 100,000 random interactions per agent). For each parameter combination (population-size and agent-composition), we repeated the simulation 50 times to obtain reliable estimates of the resulting network structure and cooperation rates across agent-types.

### Evolutionary dynamics

To analyze the success of reputation strategies, we ran evolutionary simulations. Agents were repeatedly randomly matched for *i* iterations (interaction frequencies) and accumulated payoff based on their own and their partner’s action. In each interaction, they played a prisoner’s dilemma in which they incurred a cost *c* for playing C (*x* = 1, otherwise *x* = 0), and received a benefit *b* when the partner played C (*y* = 1, otherwise *y* = 0), resulting in the following payoff function:

3 $$\begin{array}{*{20}{c}} {\pi _x = \mathop {\sum }\limits_{t = 1}^i by_t - cx_t,\,{\mathrm{where}}\,c \hskip 2pt < \hskip 2pt b} \end{array}$$

For the evolutionary simulations, we also introduced a third agent-type: the always-defect agent  (or simply defector or free-rider). The always-defect agent does not engage in relationship-scoring or updating and always chooses the selfish option D.

After the *i*th iteration, one random agent of the population was selected to adapt its strategy based on the frequency dependent Moran process with an exponential payoff function^32,62,69^. With probability *u*, the agent would adopt one of the three strategies described above with equal probability (random mutation). With probability 1 − *u*, the agent would adopt a strategy of another agent *x* in the population proportional to $e^{\pi _x}$. Strategy changes can be interpreted as either genetic evolution or social learning.

When adopting another strategy based on fitness, the probability that the number of agents with a particular strategy changes from *n* to *n* + 1 is given by:

4 $$\begin{array}{*{20}{c}} {p_{n_A \to n_{A + 1}} = \frac{{\mathop {\sum }\nolimits_{i = 1}^{n_A} e^{\pi _{Ai}}}}{{\mathop {\sum }\nolimits_{i = 1}^{n_A} e^{\pi _{Ai}} + \mathop {\sum }\nolimits_{i = 1}^{n_B} e^{\pi _{Bi}} + \mathop {\sum }\nolimits_{i = 1}^{n_C} e^{\pi _{Ci}}}}\frac{{n - n_A}}{n}} \end{array}$$

5 $$\begin{array}{*{20}{c}} {p_{n_B \to n_{B + 1}} = \frac{{\mathop {\sum }\nolimits_{i = 1}^{n_B} e^{\pi _{Bi}}}}{{\mathop {\sum }\nolimits_{i = 1}^{n_A} e^{\pi _{Ai}} + \mathop {\sum }\nolimits_{i = 1}^{n_B} e^{\pi _{Bi}} + \mathop {\sum }\nolimits_{i = 1}^{n_C} e^{\pi _{Ci}}}}\frac{{n - n_B}}{n}} \end{array}$$

6 $$\begin{array}{*{20}{c}} {p_{n_C \to n_{C + 1}} = \frac{{\mathop {\sum }\nolimits_{i = 1}^{n_C} e^{\pi _{Ci}}}}{{\mathop {\sum }\nolimits_{i = 1}^{n_A} e^{\pi _{Ai}} + \mathop {\sum }\nolimits_{i = 1}^{n_B} e^{\pi _{Bi}} + \mathop {\sum }\nolimits_{i = 1}^{n_C} e^{\pi _{Ci}}}}\frac{{n - n_C}}{n}} \end{array}$$

Likewise, the probability for an agent with strategy A to adopt strategy B or C is given by:

7 $$\begin{array}{*{20}{c}} {p_{A \to B} = \frac{{\mathop {\sum }\nolimits_{i = 1}^{n_B} e^{\pi _{Bi}}}}{{\mathop {\sum }\nolimits_{i = 1}^{n_A} e^{\pi _{Ai}} + \mathop {\sum }\nolimits_{i = 1}^{n_B} e^{\pi _{Bi}} + \mathop {\sum }\nolimits_{i = 1}^{n_C} e^{\pi _{Ci}}}}\frac{{n_A}}{n}} \end{array}$$

8 $$\begin{array}{*{20}{c}} {p_{A \to C} = \frac{{\mathop {\sum }\nolimits_{i = 1}^{n_C} e^{\pi _{Ci}}}}{{\mathop {\sum }\nolimits_{i = 1}^{n_A} e^{\pi _{Ai}} + \mathop {\sum }\nolimits_{i = 1}^{n_B} e^{\pi _{Bi}} + \mathop {\sum }\nolimits_{i = 1}^{n_C} e^{\pi _{Ci}}}}\frac{{n_A}}{n}} \end{array}$$

Supplementary Movie 4 exemplifies the change in agent composition under selection pressure in a small population of *n* = 20 agents. At the beginning, the entire population consists of defectors. Eventually, defectors are invaded by friend-focused agents that build cooperative dyadic relationships or small groups. As soon as Heider agents appear in the population, both group size (i.e., group polarization) and global cooperation rates increase. However, at this stage, the population becomes vulnerable to defectors who, eventually, take over again.

Supplementary Figure 1 shows the transition matrix based on maximum likelihood Markov chain estimations for the simulation underlying Fig. 3 (*n* = 100, 4 × 10^5^ iterations, *i* = 10, *c* = 1, *b* = 4). Mutual cooperation in the population increases when transitioning from a population of friend-focused to a population of Heider agents. However, in a population of Heider agents, there is a large likelihood of invasion by defectors, which is not the case for a population of friend-focused agents.

### Parameter space

To investigate the evolutionary dynamics across a wider parameter space, we ran simulations sampled from the parameter-space *u* ∈ {0.01, 0.001} (mutation probability), *i* ∈ {1, 2,…, 32} (interaction frequency), *b* ∈ {1, 2,…, 8} (cooperation benefit). Population size and cooperation cost was fixed to *n* = 100 and *c* = 1, respectively (resulting in the Rapoport indices of cooperation $K = \frac{{R - P}}{{T - S}}$ equal to 0, 1/3, 1/2, 2/3, 5/7, 3/4, 7/9). For each simulation, we ran *i* × 5 × 10^5^ iterations. For ease of interpretation, we aggregated data across mutation rates in the figures. Additional details are presented in Supplementary Note 1 and Supplementary Figures 3–4.

### Pairwise invasions

To understand the invasion-cycles that we observe between Heider agents, defectors, and friend-focused agents, we ran simulations of all pairwise agent combinations across the parameter space. Specifically, we analyzed friend-focused agents vs. defectors, Heider agents vs. defectors, and Heider agents vs. friend-focused agents. This allows us to investigate (a) if and when a single reputation-based agent can survive against defectors and (b) when and why Heider agents invade friend-focused agents and vice versa. Additional details are presented in Supplementary Note 2 and Supplementary Figures 5–8.

### Memory constraints

We extended our main model to impose memory constraints on the agents, by only allowing them to store *s* reputation bits in the relationship-vector **m**_*x*_ (in all other simulations *s* was equal to *n*). Each agent was able to memorize the most extreme relationships they have (i.e., their closest friends and worst enemies). In case of ties, the relationship element that the agent would forget was chosen randomly. More specifically, in each interaction, each agent has an *n*-size relationship vector for all other agents in the population based on past experience. In each step, agents forget the “weakest” relationship of the *n*–*k* agents, i.e., the *n*–*k* opinions that are closest to zero. Hence, agents forget their relationship for which they have not formed a strong “memory-trace”. The *k* strongest relationships (closest to 1 or −1, “best friends” and “worst enemies”), on the other hand, are memorized. The relationship to oneself, i.e., the diagonal of the reputation matrix was fixed to 1, as in the standard model.

We investigated the effect of memory constraint on the network structure among reputation-sensitive agents for *n* = 20, 40, 60, 120 that comprised 1, 2, 3, 4, or 5 Heider agents and a memory size of 10%, 30%, 50%, 70 and 90% of the respective group size after 10^5^ iterations. Further, we introduced two levels of memory constraints, *s* = 33 and *s* = 66, under selection pressure and ran evolutionary simulations with the parameters *n* = 100, *u* = 0.01, *b* = 4, *i* = 10, *r* = 0.3 and compared it to populations with perfect memory (see Fig. 6). To test whether the obtained results are generalizable, we further ran simulations for different parameter combinations for each memory level *s*. Additional details are presented in Supplementary Note 3 and Supplementary Figures 9–13.

### Sensitivity analyses

To further check the robustness and generalizability of the obtained results, we ran several additional simulations introducing additional agent-types, manipulating the speed at which agents form relationships, and running simulations in a larger population.

### Additional agent-types

To understand the community building properties of Heider agents that is followed by invasions of defectors, we ran simulation in which we introduced two additional agent types to further isolate the effect of specific Heider rules on cooperation, on the one hand, and the vulnerability to defectors, on the other hand.

Specifically, we define “enemy-focused agents” as agents that only take the weighted opinion of enemies into account, but do not “trust” the opinions of friends (i.e., only implement the “enemy of an enemy is a friend” and the “friend of an enemy is an enemy” heuristic). This allows us to contrast the two friend-focused Heider heuristics to the two enemy-focused Heider heuristics.

We further define “incomplete Heider agents” as agents that only implement the first three Heider heuristics (“a friend of a friend is a friend”, “an enemy of a friend is an enemy”, and “a friend of an enemy is an enemy”), but not the last heuristic (“an enemy of an enemy is a friend”). Comparing the results of Heider agents vs. incomplete Heider agents enable us to isolate the effect of the “enemy of an enemy is a friend” heuristic on population-wide cooperation and community building. Additional details are presented in Supplementary Note 4 and Supplementary Figures 14–20.

### Speed of forming relationships

The logistic decision function to cooperate or defect of our model (Eq. (2)) is illustrated in Supplementary Figure 2. Each value in the reputation matrix **S** was bound to lie between −1 and 1, which corresponds to a probability close to 0 and 1 to not cooperate (defect) or cooperate, respectively. This boundary was chosen because values above 1 or below −1 would not significantly alter the probability of actions (C vs. D). At the same time, it gives the fixed value of the relationship every agent has with itself (*s*_*xx*_) an intuitive meaning: The relationship to another agent is bound to be worse or as good as the relationship that the agent has to itself. For reputation, this means that an agent can trust the opinion of another agent as much as the agent would trust its own opinion, but not more.

The temperature parameter of the logistic function, the boundaries, and the change in opinion/relationship *r* based on the action of the opponent, together, determine how fast an agent is building a relationship with another agent and switches from defection to cooperation or vice versa. Hence, these three parameters determine how forgiving or punishing an agent is. The main analysis was performed with *r* = 0.3. To see how the population dynamics change when agents are less or more forgiving (hence, form relationships slower or faster), we further ran simulations with *r* = 0.1 and *r* = 0.5, sampling across the full parameter space. With *r* = 0.1, agents with a neutral opinion would increase their likelihood to cooperate (defect) from *p* = 0.5 to *p* = 0.62 after an interaction (solely based on their own relationship). With *r* = 0.5, on the other hand, agents with a neutral opinion would increase their likelihood to cooperate (defect) from *p* = 0.5 to *p* = 0.92 after an interaction (solely based on their own relationship). Note that changing the value *r* is analogous to changing the temperature parameter of the logistic function. By increasing (decreasing) *r*, the decision function becomes steeper (flatter), meaning that fewer interactions are needed to establish a positive or negative relationship (Supplementary Figure 2b). Additional details are presented in Supplementary Note 5 and Supplementary Figures 21–22.

### Larger population

Our main evolutionary simulations use a population-size of *n* = 100, thereby approximating the size of social friendship networks^70–72^ or international alliances^73,74^. Interestingly, the degree distribution of social networks is usually not normally distributed but follows a power law or log-normal distribution (e.g., ref. ^72^). This resonates with our network structure and degree distribution that we observe in a population comprised of a majority of friend-focused agents and a minority of Heider agents.

Small populations are more influenced by the stochasticity of the Moran process making it easier for neutral drifts to occur. To check the robustness of the results, in particular the dynamic shifts of agent-compositions and group fission–fusion dynamic, we repeated the simulations with a larger population (*n* = 500) sampling from the full parameter space (*u* ∈ {0.01, 0.001}, *i* ∈ {1, 2, …, 32}, *b* ∈ {1, 2,…, 8}, and *r* ∈ {0.1, 0.3, 0.5}). Additional details are presented in Supplementary Note 6 and Supplementary Figures 23–24.

### Code availability

The code used for data analysis and simulations is available from the corresponding author upon reasonable request.

## Supplementary information

Supplementary Information

Description of Additional Supplementary Files

Supplementary Movie 1

Supplementary Movie 2

Supplementary Movie 3

Supplementary Movie 4

## Data Availability

The data that support the findings of this study are available from the corresponding author upon reasonable request.
