# Supplementary material for: The rise and fall of cooperation through reputation and group polarization
Source: Nat Commun. 2019 Feb 15;10:776. doi: 10.1038/s41467-019-08727-8 (PMC6377668; doi:10.1038/s41467-019-08727-8)
Supplement: Supplementary file 1 — Supplementary Information [file 41467_2019_8727_MOESM1_ESM.pdf]

Supplementary Information

The rise and fall of cooperation through reputation and group polarization

Gross & De Dreu

Supplementary Figures 2

Supplementary Note 1 3

Supplementary Note 2 5

Supplementary Note 3 10

Supplementary Note 4 13

Supplementary Note 5 20

Supplementary Note 6 23

Supplementary Figures

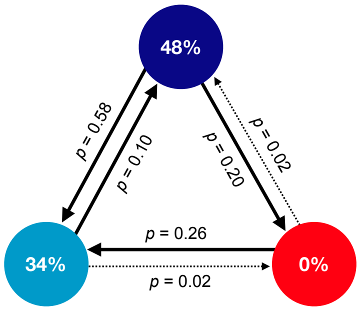

Supplementary Figure 1. State transitions. Probabilities to transition from a population of a majority (>50%) of one type of agent to another type of agent (dark blue circle = Heider population, light blue circle = friend-focused population, red circle = free-rider population). Numbers in circles represent the average mutual cooperation in a majority state of the respective agent type. Based on $n = 100$, 4×10^5 iterations, $i = 10$, $c = 1$, $b = 4$.

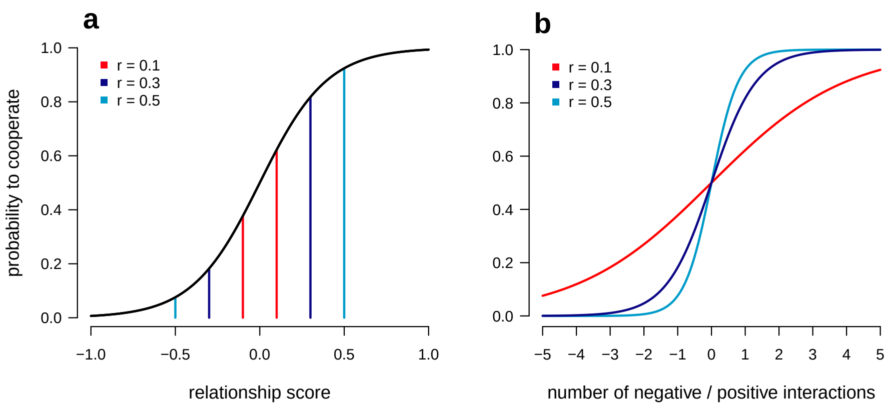

Supplementary Figure 2. Decision function. Probability to cooperate based on the relationship score (a). Vertical lines indicate different values for the relationship change r in the model (red line: $r = 0.1$, dark blue line: $r = 0.3$, light blue line: $r = 0.5$). By changing r, agents need more or less interactions to establish a positive or negative relationship, essentially changing the steepness of the decision function (b).

Supplementary Note 1

Supplementary Figure 3 shows the average expected proportion of each agent depending on the benefit of cooperating b and the number of interactions i before an adaptation takes place (aggregated across mutation rates), based on best linear fit. When the cooperation benefit is low and there are frequent adaptations, the population spends most of its time in a homogenous state consisting of defectors only. With increased cooperation benefit and/or frequent interactions, the relative proportion of defectors in the population declines (Supplementary Figure 3a, linear regression; β_b = -8.78, β_i = -0.67, $\beta_b \times \beta_i$ = -0.01). A higher benefit of cooperation and more frequent interactions increase the proportion of friend-focused agents (Supplementary Figure 3b, linear regression; $\beta_b = 5.42$, $\beta_i = 0.52$, $\beta_b \times \beta_i$ = -0.04). Interestingly, the proportion of Heider agents stays relatively constant and only increases significantly with very frequent interactions and/or very high return on cooperation (Supplementary Figure 3c, linear regression; $\beta_b = 3.36$, $\beta_i = 0.14$, $\beta_b \times \beta_i = 0.05$).

Heider agents are in the minority across all parameter combinations. On average 19% of the agents in the population are Heider agents. The proportion of Heider agents relative to the proportion of all reputation sensitive agents in the population (i.e. the proportion of Heider and friend-focused agents) is largely independent of the parameter-configuration (Supplementary Figure 4a, linear regression; $\beta_b = 0.02$, $\beta_i = 0.002$, $\beta_b \times \beta_i = 0.0003$). Hence, there is a stable but low proportion of Heider agents across the parameter space (except for very low interaction frequency and/or return on cooperation). Alongside the decline of defectors in the population, mutual cooperation rates consequently increase by a factor of 4% for every unit increase in b and 0.4% for every unit increase in i (Supplementary Figure 4b, linear regression; $\beta_b = 0.04$, β_i = 0.004, $\beta_b \times \beta_i = 0.0002$).

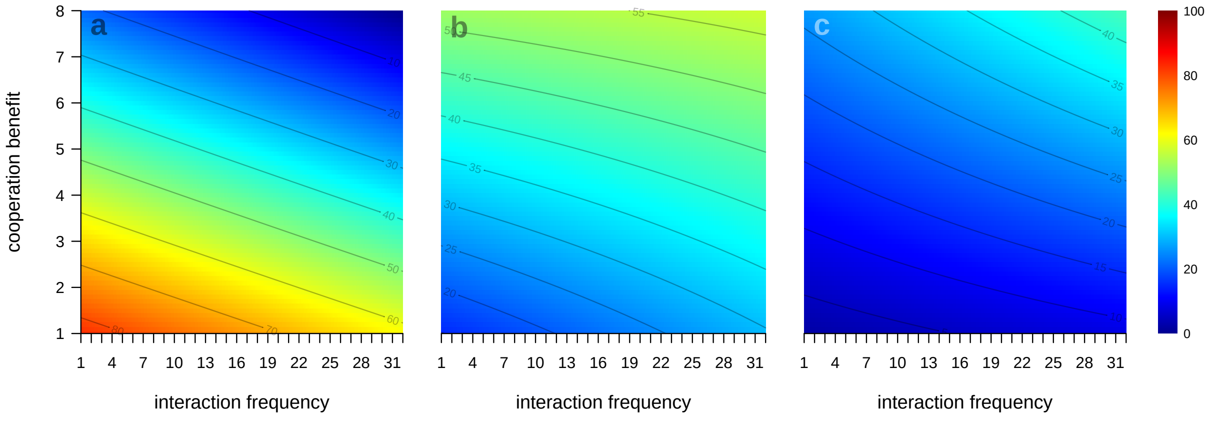

Supplementary Figure 3. Population composition across the parameter space. Estimated percentage of defectors (a), friend-focused agents (b), and Heider agents (c) as a function of the return on cooperation b and the interaction frequency i before adaptation takes place.

We further calculated the variance of the proportion of the three types for each parameter set across time. This serves as a proxy for the instability of the population by estimating how fast the population iterates between different states (speed of state-transition dynamic). Supplementary Figure 4c shows that a combination of low interaction-frequency and/or low cooperation benefit leads to a relatively stable population state, due to the dominance of defectors. Increased interaction-frequency and benefit on cooperation increases cooperation, but also leads to more instability in the populations with more rapid changes in the population's agent-composition, accompanied by a rise and fall of cooperative communities (linear regression; $\beta_b = 2.68$, $\beta_i = 0.28$, $\beta_b \times \beta_i =$ -0.02).

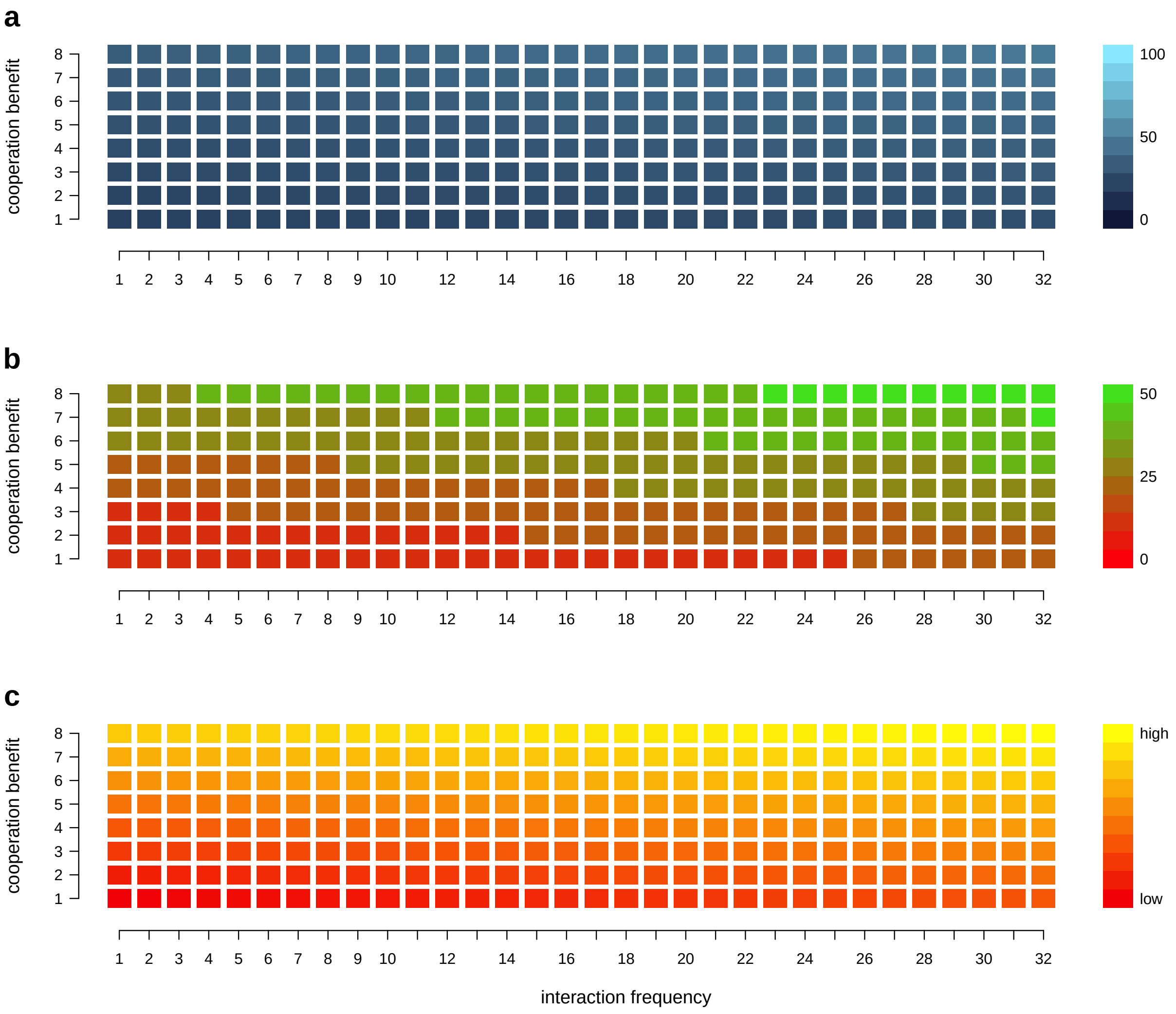

Supplementary Figure 4. Population characteristics across the parameter space. Panels show the relative proportion of Heider agents among Heider and friend-focused agents (a), average frequency of mutual cooperation (b), and population instability, calculated by the variance in the agent-composition across time (c), depending on return on cooperation (benefit b) and interaction frequency.

Supplementary Note 2

To understand the pairwise invasion dynamic of the three agent-types, we ran simulations with populations that only consists of (a) defectors and friend-focused agents, (b) defectors and Heider agents, and (c) friend-focused and Heider agents.

Defectors vs. friend-focused agents

Supplementary Figure 5 shows that friend-focused agents were successful against defectors, even more so than in the three-type simulations. Already with low interaction frequency and return on cooperation they make up the majority of the population (linear regression; β_b = 11.48, β_i = 0.49, $\beta_b \times \beta_i$ = -0.06). This apparent advantage of friend-focused agents against defectors also leads to more stable populations, especially with increased cooperation returns (Supplementary Figure 6b, linear regression; β_b = -1.56, β_i = -0.26 , $\beta_b \times \beta_i$ = 0.03).

However, the dominance of friend-focused agents in the population does not translate to high cooperation rates. Across the entire parameter space, mutual cooperation is rare and does not exceed 11% of the interactions (Supplementary Figure 6a, linear regression; β_b = 0.008, β_i = 0.0004 , $\beta_b \times \beta_i$ = 0). This is due to scattered groups that cooperate within, but not between group members. On average, the population consists of 35 independent groups that do not share any cooperative connections between each other. Hence, without Heider agents, the population is stable and is comprised of mostly friend-focused agents. Without Heider agents, population-wide cooperation rates are much lower.

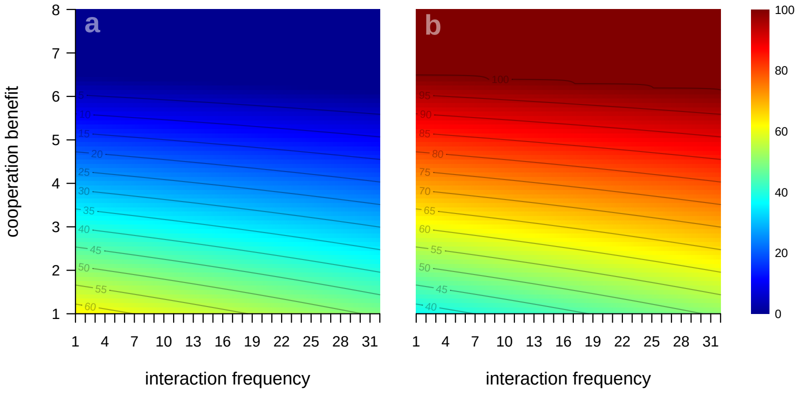

Supplementary Figure 5. Population composition in the defector vs. friend-focused agents simulations. Estimated percentage of defectors (a) and friend-focused agents (b) as a function of the return on cooperation b and the interaction frequency i before adaptation takes place.

Defectors vs. Heider agents

As Heider agents treat an enemy of an enemy as a friend, they have a high likelihood to cooperate with out-group members. While in combination with friend-focused agents, this characteristic helps to make connections with other groups, they are unable to systematically isolate defectors and instead may cooperate with defectors (since they likely have a considerable number of common enemies). The full Heider reputation principles, based on psychological transitivity

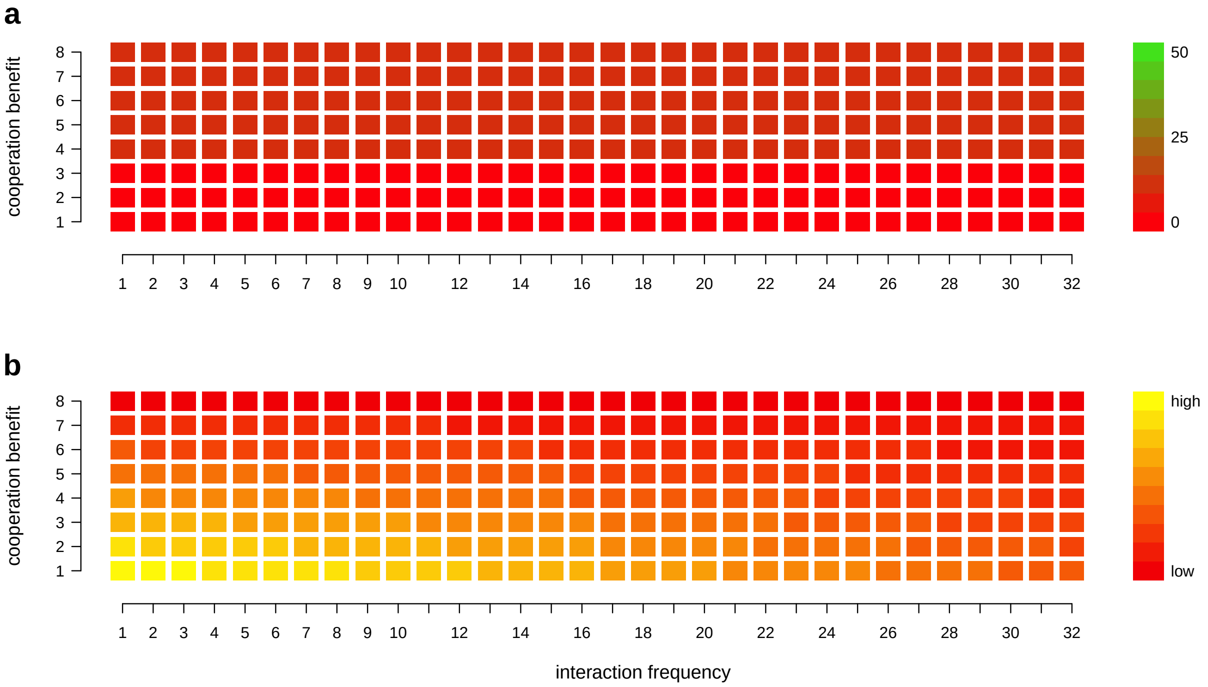

Supplementary Figure 6. Population characteristics in the defector vs. friend-focused agents simulations. (a) Average frequency of mutual cooperation and (b) population instability, depending on return on cooperation (benefit b) and interaction frequency.

and psychological balance [1], are hence highly exploitable by free-riders. Consequently, Heider agents, in stark contrast to friend-focused agents, do not survive against defectors across the full parameter space. Defectors dominate the population (Supplementary Figure 7) and cooperation is not sustainable.

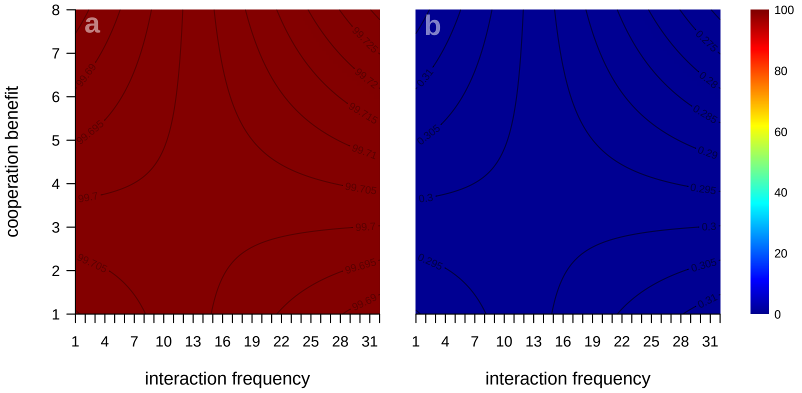

Supplementary Figure 7. Population composition in the defector vs. Heider agents simulations. Estimated percentage of defectors (a) and Heider agents (b) as a function of the return on cooperation b and the interaction frequency i before adaptation takes place.

Friend-focused vs. Heider agents

As can be seen in Figure 5 of the main manuscript, the two additional enemy heuristics that Heider agents, but not friend-focused agents, use lead to more positive outgoing connections.

This difference in connectedness is accompanied by a relative fitness advantage when Heider agents are in the minority. With an increase in Heider agents in the population, the difference in connectedness and, consequently, the fitness advantage of Heider agents over friend-focused agents disappears.

This initial advantage of Heider agents over friend-focused agents is due to the polarization effect of Heider agents. In a network that is comprised of multiple isolated communities (e.g. Figure 1b in the main manuscript), the friend-focused heuristics ('the friend of my friend is my friend' and 'the enemy of my friend is my enemy') do not allow agents to establish new connections to other groups, once every community is balanced (i.e. every friend is also a friend of my friends and every enemy is also an enemy of my friends). Heider agents, on the other hand, can make connections to agents of other groups as long as there are more than two groups in the population.

To understand why, assume there are several groups that are perfectly interconnected (every friend of a friend is a friend) but share only negative connections between groups (every enemy of a friend is an enemy). A reputation-sensitive agent that belongs to group A will *decrease* the likelihood to cooperate with a member from group B relative to the number of group members n_A due to 'the enemy of a friend is an enemy' heuristic. Heider agents and friend-focused agents do not differ in this respect. A Heider agent that belongs to group A will further *decrease* the likelihood to cooperate with a member from group B relative to the number of group members n_B due to 'the friend of an enemy is an enemy' heuristic. However, a Heider agent that belongs to group A will *increase* the likelihood to cooperate with a member from group B relative to the number of agents that do not belong to group A nor group B (denoted as n_D) due to 'the enemy of an enemy is a friend' heuristic. This leads to:

$$\begin{gathered}\mathrm{rs} = n_D - (n_A + n_B), \text{ where } A \cap B = B \cap D = D \cap A = \varnothing \\ \text{and} \\ p(C|x \in A) > 0.5 \iff n_D > n_A + n_B\end{gathered} \tag{1}$$

In other words, when every community is balanced (i.e. every friend of a friend is a friend and every enemy of a friend is an enemy), a Heider agent x_A will cooperate with an agent y_B with $p > 0.5$, if x and y share more enemies (i.e. agents that do not belong to A nor B; n_D) than the number of friends of x (n_A) and the number of friends of y (n_B). This is also the reason why Heider agents, but not friend-focused agents, can have a positive likelihood to cooperate with isolated defectors, since Heider agents and defectors can share the same enemies (n_D), while isolated defectors have no friends, except for themselves ($n_B = 1$).

Consequently, we observe that Heider agents have more positive outgoing connections the more communities exist in the population. The number of communities, i.e. the modularity of the network, decreases as the proportion of Heider agents in the population increases – Heider agents polarize the network structure. As soon as the network is maximally polarized (e.g. Figure 1a in the main manuscript), the network enters the Heider equilibrium: Every member of a group shares the same friends and the same enemies. With two densely interconnected groups, there are no enemies of enemies anymore. In this state, Heider agents and friend-focused agents become indistinguishable in their behaviour.

In the simulations underlying Figure 5 in the main manuscript we, accordingly, observe that with increased polarization of the network (Figure 5b), the relative fitness advantage of Heider

agents over friend-focused agents disappears (Figure 5d) which allows a neutral drift from a population of Heider agents to a population of friend-focused agents. Heider agents can invade friend-focused agents and vice versa, leading to the cycles we observe in Figure 5a.

The relative frequency of Heider agents in the population should depend on the cooperation benefit b and the interaction frequency i. With higher cooperation benefit b, the return of more (positive) connections is higher, giving Heider agents a greater advantage over friend-focused agents, when in the minority. Additionally, higher interaction frequency i gives Heider agents more time to establish and profit from these connections before a mutation occurs.

In line with these hypotheses, we observe that the relative proportion of Heider agents increase with b and i (Supplementary Figure 8a, linear regression, β_b = 4.6, β_i = 1.6, $\beta_b \times \beta_i$ = -0.03).

Since Heider agents become indistinguishable from friend-focused agents once the population network gets closer to the Heider equilibrium, we never observe that Heider agents dominate friend-focused agents across the full parameter space. However, in an environment with a low return on cooperation b and low interaction frequency i, the initial fitness advantage of Heider agents is lower and prevents Heider agents to spread. This is exemplified in Supplementary Figure 8b and Supplementary Figure 8c, comparing the population dynamic in an environment with low return of cooperation and low interaction frequency ($b = 2$, $i = 4$) with an environment with high return of cooperation and high interaction frequency ($b = 7$, $i = 28$). In both situations, we observe the same general pattern: Heider agents in the minority have more (positive) out-going connections and a fitness advantage over friend-focused agents. However, under low b and i, the network does not come close to the Heider equilibrium due to frequent mutations and strategy shifts and the relative fitness advantage of Heider agents is lower due smaller returns of cooperation.

More generally, we find that the number of communities decrease (i.e. polarization increases) the more frequently agents interact before a mutation takes place (linear regression, β_b = -0.35 , β_i = -0.77, $\beta_b \times \beta_i$ = 0.01) and the percentage of mutually cooperative interactions increase with b and i (linear regression, β_b = 0.86, β_i = 0.36, $\beta_b \times \beta_i$ = -0.03).

Summary

The above patterns can be summarised as follows:

(1) Heider agents, on their own, are not successful against defectors.
(2) Friend-focused agents are successful against defectors but cooperation-rates stay low.
(3) Heider agents can invade friend-focused agents.
(4) Heider agents increase population-wide cooperation by increasing group size.
(5) With increased polarization (i.e. decrease in number of communities in the network), Heider agents become indistinguishable from friend-focused agents in their fitness, connectedness, and behaviour.
(6) However, Heider agents can make a population vulnerable to defectors.
(7) Defectors can successfully invade whenever a population is highly polarized and consists of many Heider agents.

Heider agents are, thus, a double-edged sword. They polarize the population, i.e. decrease the number of communities, thereby increasing population-wide cooperation. At the same time, once they reach a majority in the population, they can be invaded by defectors.

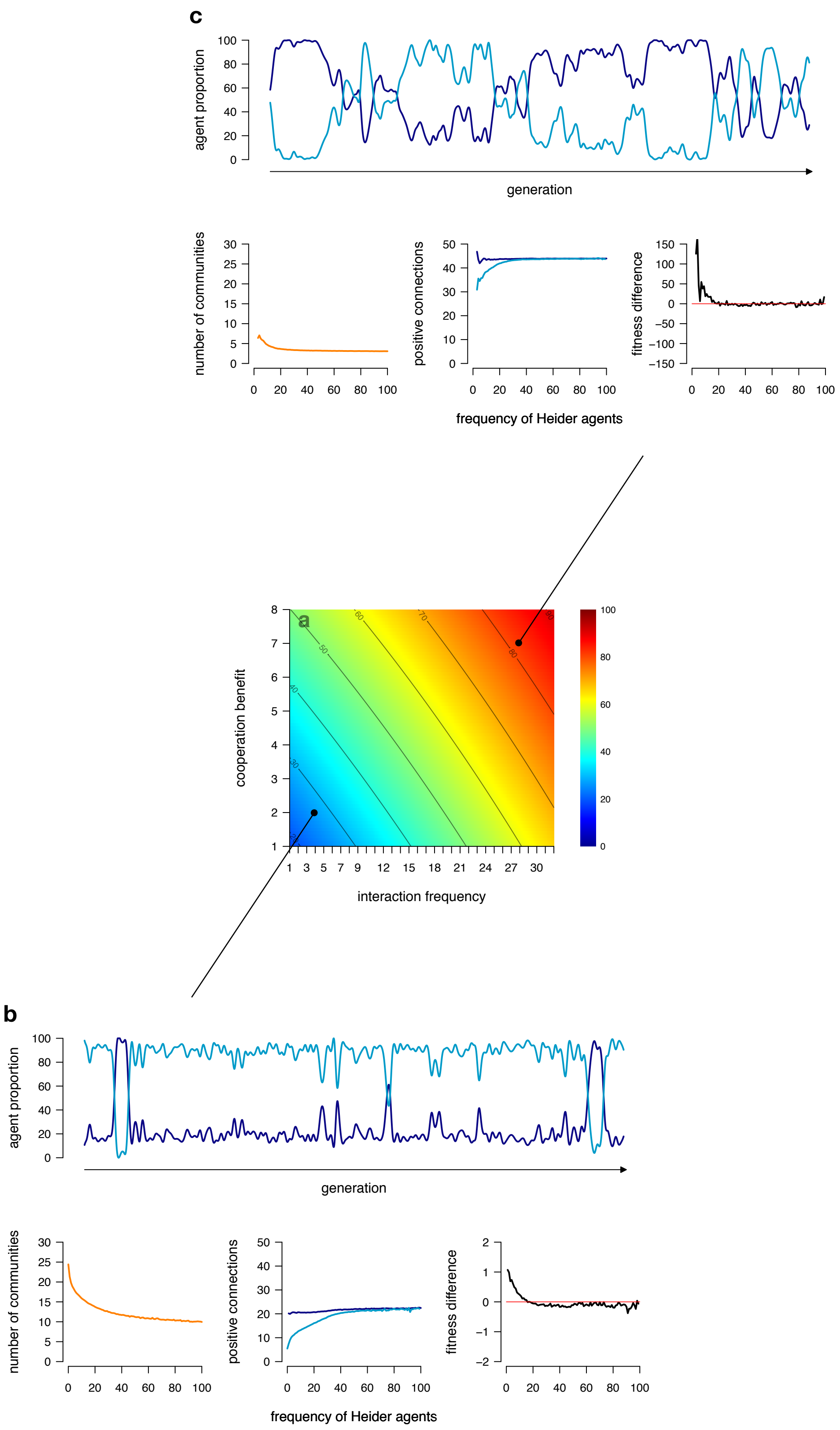

Supplementary Figure 8. Estimated percentage of Heider agents in a population of friend-focused and Heider agents as a function of the return on cooperation b and the interaction frequency i before adaptation takes place (a). Panel b and c exemplify the population dynamic, the community structure, number of positive out-going connections for Heider agents (dark blue line) and friend-focused agents (light blue line), and fitness difference between Heider agents and friend-focused agents (positive values indicate higher relative fitness of Heider agents) for $b = 2$, $i = 4$ (b) and $b = 7$, $i = 28$ (c).

Supplementary Note 3

Figure 6a in the main manuscript shows the number of positive connections between agents across a population size of $n = 10$, 20, 40, 60, and 120 aggregated across 0 to 5 Heider agents in the population with increased memory capacity. Supplementary Figure 9 shows this result across the different number of Heider agents that were simulated. On average, positive connections between agents increase with increased memory capacity (linear regression; memory $\beta_s = 3.13$). This increase was moderated by the number of Heider agents. The more Heider agents in a population, the more groups benefitted from higher memory capacity (linear regression; $\beta_s \times \beta_{\text{Heider agents freq.}} = 1.06$).

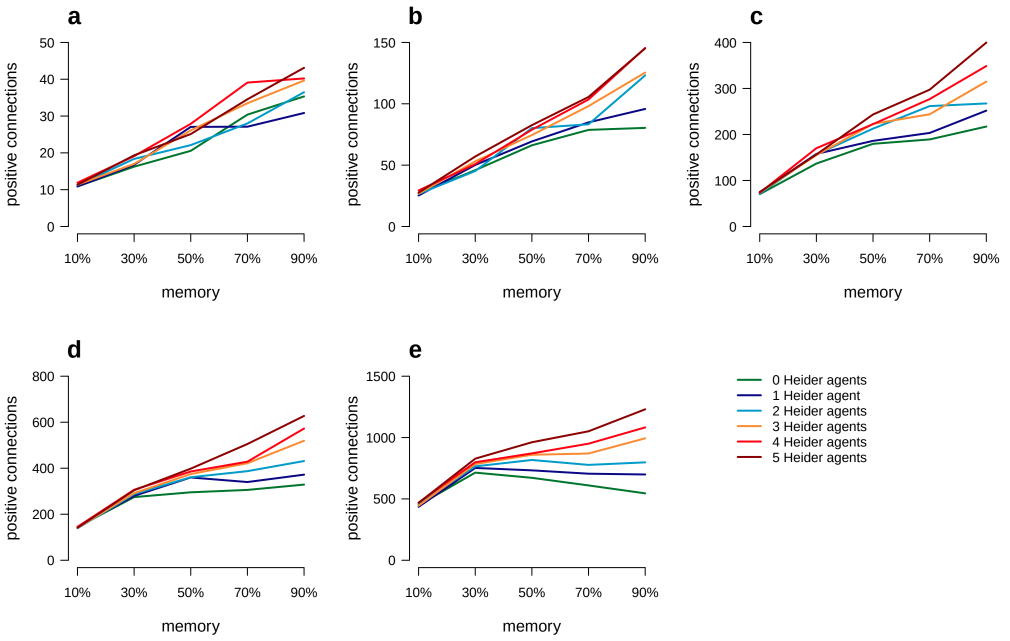

Supplementary Figure 9. Network connectedness based on memory size and number of Heider agents (green line: 0 Heider agents, dark blue line: 1 Heider agent, light blue line: 2 Heider agents, yellow line: 3 Heider agents, light red line: 4 Heider agents, dark red line: 5 Heider agents) across a population of (a) $n = 10$, (b) $n = 20$, (c) $n = 40$, (d) $n = 60$, (e) $n = 120$.

This acceleration of positive connections as a function of memory capacity in interaction with Heider agents consequently increases the success of Heider agents under selection pressure (Figure 6b in the main manuscript). However, with increased number of Heider agents, we observe more rapid cycles between pure defection, populations of friend-focused agents, and populations of Heider agents. Hence, populations become more unstable and the oscillation speed between group emergence and collapse of cooperation increases. This can also be seen in the increased likelihood of transitioning from friend-focused populations to Heider populations in the empirically estimated Markov chain transition probabilities that results from increased memory capacity (Supplementary Figure 10).

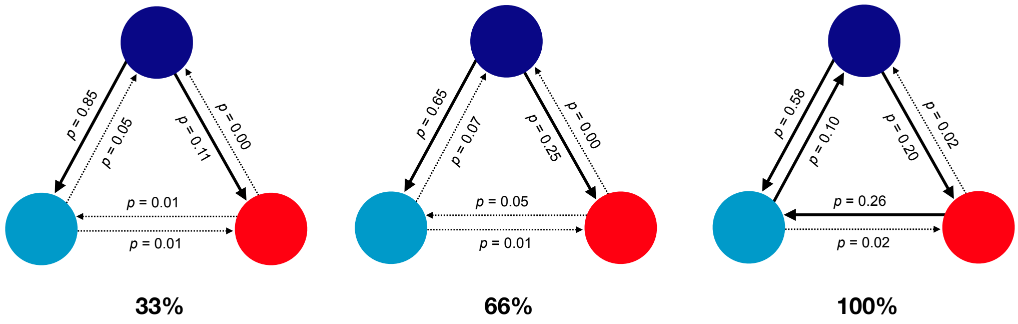

Supplementary Figure 10. State transitions across memory capacities. Probabilities to transition from a population of a majority (>50%) of one type of agent to another type of agent (dark blue circle = Heider population, light blue circle = friend-focused population, red circle = free-rider population) based on $n = 100$, 4×10^5 iterations, $i = 10$, $c = 1$, $b = 4$.

These results generalize to the full parameter space. Higher information transmission or higher memory capacity increased the average number of positive connections in the population in interaction with cooperation benefit and interaction frequency (Supplementary Figure 11, linear regression, $\beta_{\text{memory}} \times \beta_i = 0.56$, $\beta_{\text{memory}} \times \beta_b = 2.32$).

With higher memory capacity, the relative proportion of Heider agents in the population increase in interaction with cooperation benefit and interaction frequency (Supplementary Figure 12, linear regression, three-way interaction; $\beta_{\text{memory}} \times \beta_i \times \beta_b = 0.004$).

While higher memory capacity leads to more positive connections, the populations are more unstable across time due to a higher proportion of Heider agents. Supplementary Figure 13 shows the average network instability, measured by the variance in the agent-composition across time for the different memory levels and other parameters. Instability increases with larger memory (linear regression, $\beta_{\text{memory}} = 0.12$, controlling for all other parameters and interactions).

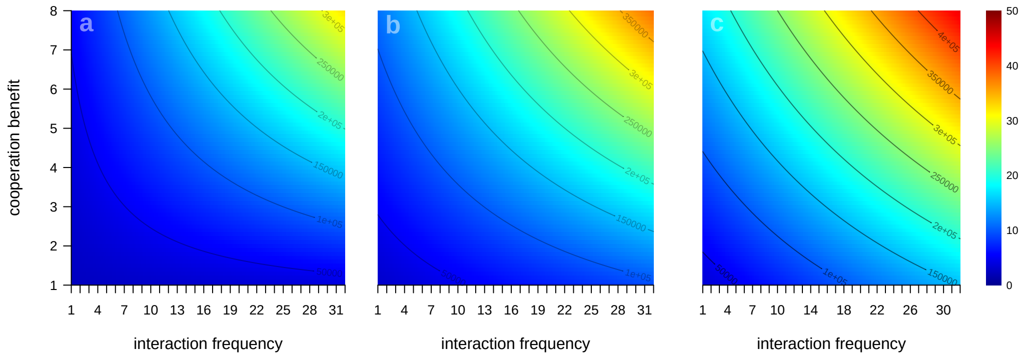

Supplementary Figure 11. Network connectedness across parameter space. Estimated number of average positive network connections for low (a), medium (b), and high (c) memory capacity, depending on the return on cooperation and interaction frequency.

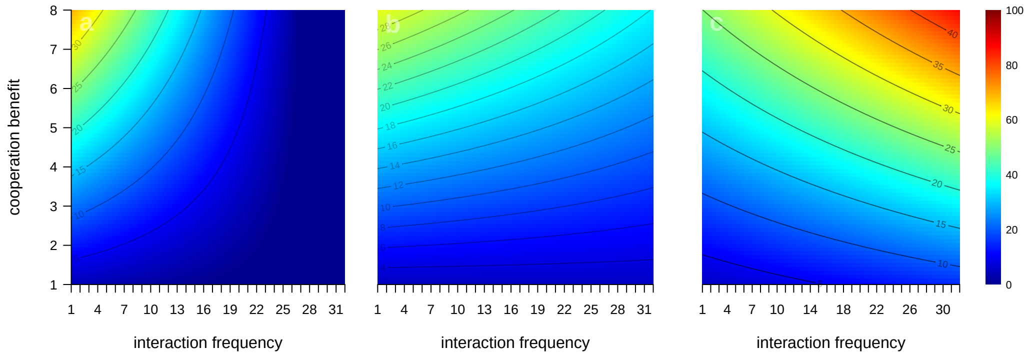

Supplementary Figure 12. Proportion of Heider agents across parameter space. Estimated percentage of Heider agents in the population for low (a), medium (b), and high (c) memory capacity, depending on the return on cooperation and interaction frequency.

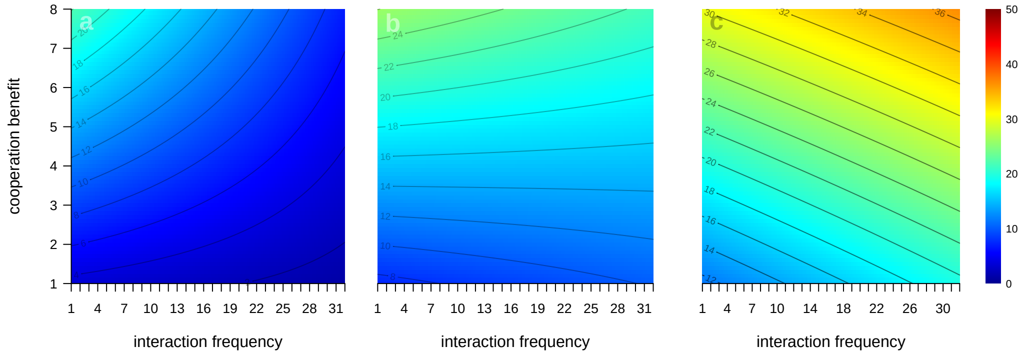

Supplementary Figure 13. Network instability across parameter space. Instability, calculated by the variance in the agent-composition across time for low (a), medium (b), and high (c) memory capacity, depending on the return on cooperation and interaction frequency.

Supplementary Note 4

To understand the community building properties of Heider agents that is followed by invasions of defectors, we ran simulation in which we introduced two additional agent types to further isolate the effect of specific Heider rules on cooperation, on the one hand, and the vulnerability to defectors, on the other hand.

Enemy-focused agents. 'Enemy-focused agents' only take the weighted opinion of enemies into account, but do not 'trust' the opinions of friends (i.e. only implements the 'enemy of an enemy is a friend' and the 'friend of an enemy is an enemy' heuristic). This allows us to contrast the two friend-focused Heider heuristics to the two enemy-focused Heider heuristics.

Incomplete Heider agents. 'Incomplete Heider agents' only implement the first three Heider heuristics ('a friend of a friend is a friend', 'an enemy of a friend is an enemy', and 'a friend of an enemy is an enemy'), but not the last heuristic ('an enemy of an enemy is a friend'). Comparing results of Heider agents vs. incomplete Heider agents enable us to isolate the effect of the 'enemy of an enemy is a friend' heuristic on population-wide cooperation and community building.

Supplementary Table 1 summarises the four reputation-based agents for the additional agent-types analyses. Across these four agent-types, we first ran simulations without strategy adaptation to compare the emerging network structure when agents are not under selection pressure (see below).

Supplementary Table 1. Agent-types based on Heider rules.

**agent label**	E_1	E_2	F_1	F_2
● Heider agent	×	×	×	×
● incomplete Heider agent	-	×	×	×
● friend-focused agent	-	-	×	×
● enemy-focused agent	×	×	-	-

E_1 = 'the enemy of my enemy is my friend'
E_2 = 'the friend of my enemy is my enemy'
F_1 = 'the enemy of my friend is my enemy'
F_2 = 'the friend of my friend is my friend'

Network structure

We first compared the network structure that emerges for the four reputation heuristics when agents do not switch strategies based on fitness.

Supplementary Figure 14 shows examples of the emerging network structure in a population of Heider agents (Supplementary Figure 14a), friend-focused agents (Supplementary Figure 14b), enemy-focused agents (Supplementary Figure 14c, 'the enemy of my enemy is my friend', 'the friend of my enemy is my enemy'), and incomplete Heider agents (Supplementary Figure

14d, 'the friend of my enemy is my enemy', 'the enemy of my friend is my enemy', 'the friend of my friend is my friend'). Agents that implement 'the enemy of my enemy is my friend' heuristic converge to two large opposing groups, that are densely interconnected within, but share no positive connections between groups. Agents that do not implement 'the enemy of my enemy is my friend', on the other hand, build smaller, more scattered communities.

Supplementary Figure 15a shows that Heider agents and enemy-focused agents significantly decrease the number of communities in the population, leading to higher mutual cooperation rates (Supplementary Figure 15b). Incomplete Heider agents, on the other hand, build more scattered communities than friend-focused agents, leading to the lowest cooperation rates (Supplementary Figure 15b) of all agents, due to the 'the friend of my enemy is my enemy' heuristic that sets them apart from friend-focused agents.

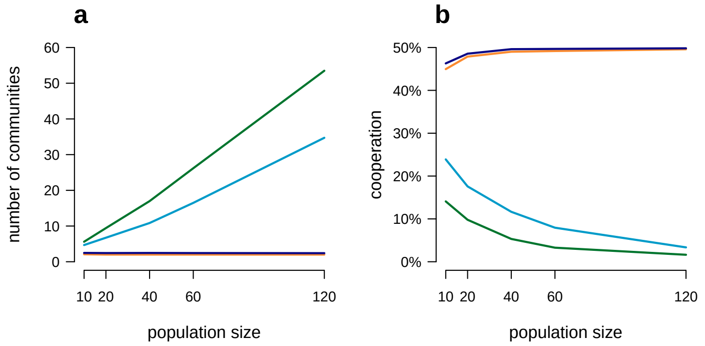

Supplementary Figure 15. Number of communities (a, measured by the Louvain method for community detection [2]) and average mutual cooperation rates (b) after 10^5 iterations across different population sizes ($n = \{10, 20, 40, 60, 120\}$) in a population of Heider agents (dark blue line), friend-focused agents (light blue line), enemy-focused agents (orange line), and incomplete Heider agents (green line).

Enemy-focused agents vs. defectors

The Heider agents implement the full Heider heuristics (hence the friend-part and the enemy-part). As shown in the pairwise invasion simulations (Supplementary Note 2), agents that only implement the friend-part of the Heider heuristics (friend-focused agents) can survive against defection, while the full implementation is not able to survive in any of the observed parameter combinations. To see whether the low survival rate of the Heider heuristics is actually due to the enemy heuristics, we ran simulations of enemy-focused agents against defectors.

The results of defectors vs. enemy-focused agents mimic the results of defectors vs. Heider agents. Enemy-focused agents do not survive against defectors across the full parameter space. Defectors dominate the population (Supplementary Figure 16) and cooperation is not sustainable, showing that the enemy-part of the Heider heuristics is responsible for the invadability by defectors.

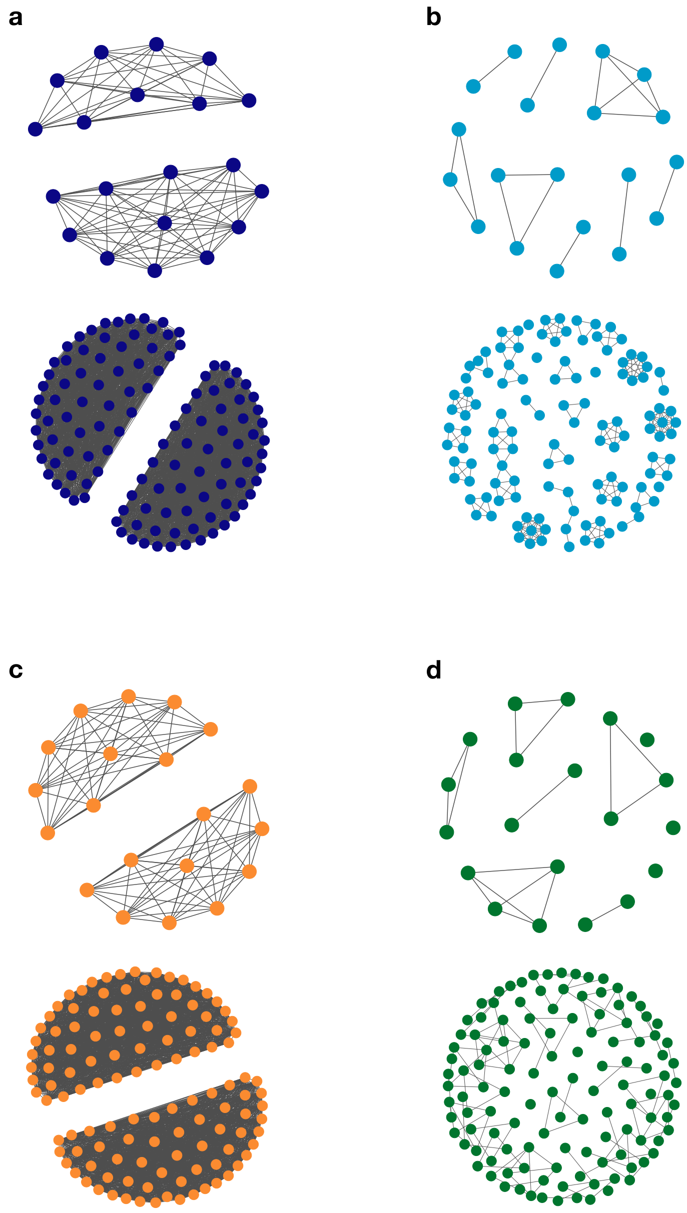

Supplementary Figure 14. Emerging community structure after 10^5 iterations in small ($n = 20$) and large ($n = 120$) populations of (a) Heider agents, (b) friend-focused agents, (c) enemy-focused agents, and (d) incomplete Heider agents. Links between agents represent a positive relationship.

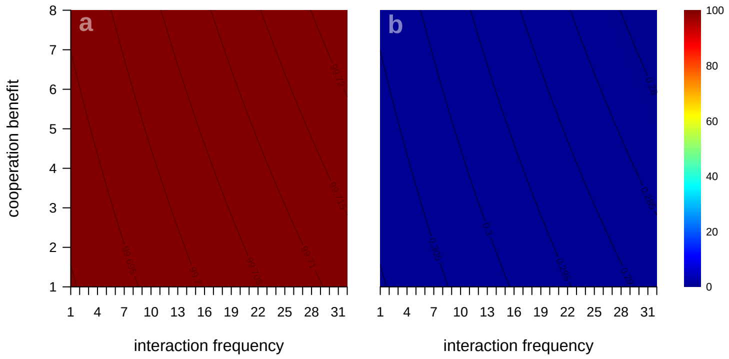

Supplementary Figure 16. Population composition in the defector vs. enemy-focused agents simulations. Estimated percentage of defectors (a) and enemy-focused agents (b) as a function of the return on cooperation b and the interaction frequency i before adaptation takes place.

Incomplete Heider agents

Defectors invade Heider agents due to the enemy-part of the Heider heuristic, yet the two enemy-heuristics ('the friend of my enemy is my enemy' and 'the enemy of my enemy is my friend') are also responsible for the increase in cooperation in a mixed population of friend-focused and Heider agents.

As shown in Supplementary Figure 14, incomplete Heider agents create a similar network structure as friend-focused agents with small cooperative clusters. We therefore expected that incomplete Heider agents can survive against defectors but show low population-wide cooperation.

As can be seen in Supplementary Figure 17, incomplete Heider agents indeed survive against defectors when the cooperation benefit and interaction frequency is high enough (linear regression, β_b = 11.48, β_i = 0.49, $\beta_b \times \beta_i$ = -0.06). To survive against defectors incomplete Heider agents need a higher return on cooperation than friend-focused agents (linear regression, $\beta_b \times \beta_{\text{friend-focused}} = 5.36$, i.e. for every one-point increase in b, we observe 5.4% more friend-focused agents than incomplete Heider agents when competing with defectors).

Like with friend-focused agents, incomplete Heider agents can survive against defectors but only exhibit low levels of population-wide cooperation (Supplementary Figure 18, linear regression, $\beta_b = 0.008$, $\beta_i = 0.0004$, $\beta_b \times \beta_i$ = -0.0001). The level of cooperation is significantly lower compared to friend-focused agents (linear regression, $\beta_{\text{friend-focused}} = 0.03$, $\beta_{\text{friend-focused}} \times \beta_b = 0.005$, i.e. we observe 3% more mutual cooperation across the parameter space and 0.5% more mutual cooperation with every point increase in b in the simulations with friend-focused agents).

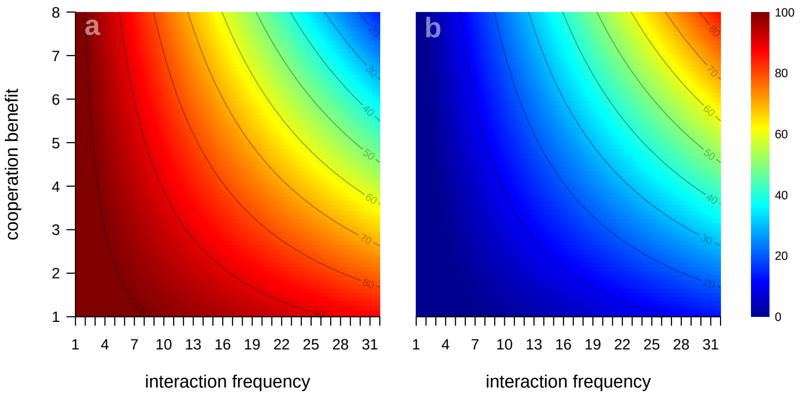

Supplementary Figure 17. Population composition in the defector vs. incomplete Heider agents simulations. Estimated percentage of defectors (a) and incomplete Heider agents (b) as a function of the return on cooperation b and the interaction frequency i before adaptation takes place.

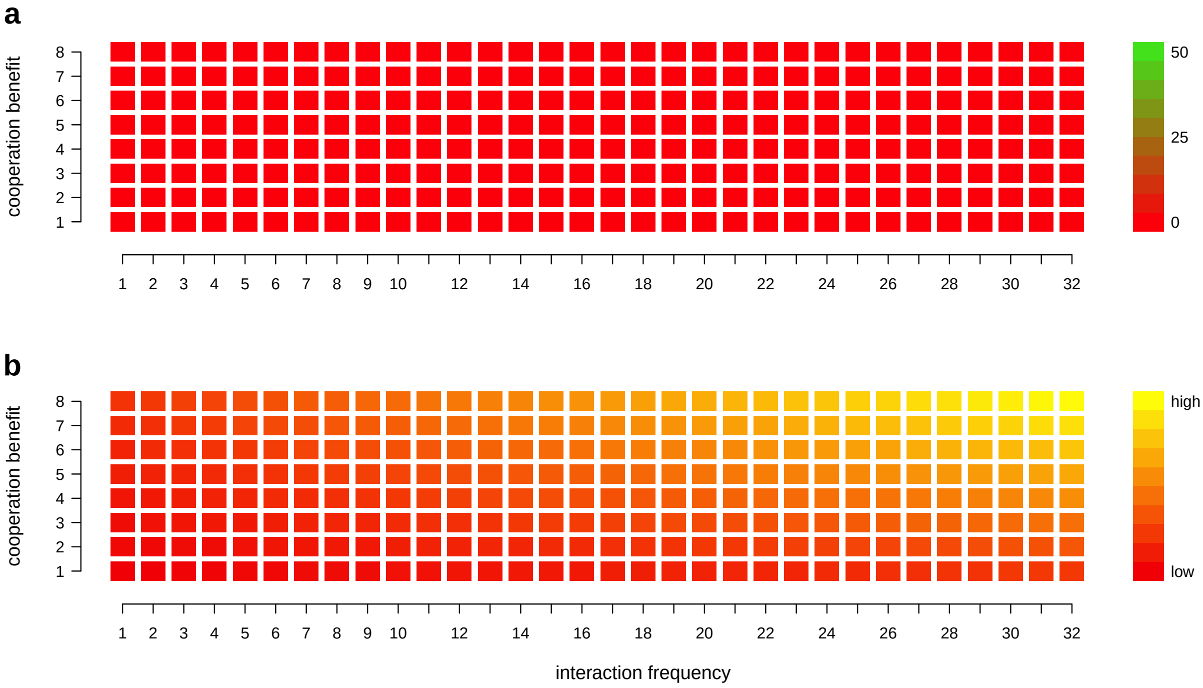

Supplementary Figure 18. Population characteristics in the defector vs. incomplete Heider agents simulations. (a) Average frequency of mutual cooperation and (b) population instability, depending on return on cooperation (benefit b) and interaction frequency.

We further ran evolutionary simulations of defectors, incomplete Heider agents, and friend-focused agents to compare the evolutionary dynamic to our main simulations. Supplementary Figure 19 shows the proportion of friend-focused agents, incomplete Heider agents, and defectors across the parameter space. As in the simulations with Heider agents, incomplete Heider agents stay largely in the minority (Supplementary Figure 20a). Contrary to the simulation with Heider agents, we observe low levels of population-wide cooperation (Supplementary Figure 20b).

Cooperation rates even decrease compared to our simulation with friend-focused agents and

defectors (linear regression, $\beta_{\text{incompl.Heider}}$ = -0.03, i.e. across the parameter space, introducing incomplete Heider agents to a population of friend-focused agents and defectors decreases average cooperation by 3%). Incomplete Heider agents, contrary to Heider agents, do not help friend-focused agents, as they lack the ability of Heider agents to establish larger group-structures ('the enemy of my enemy is my friend'). Instead, incomplete Heider agents increase the modularity of the network, due to the 'the friend of my enemy is my enemy' heuristic (see also Supplementary Figure 14d and 15).

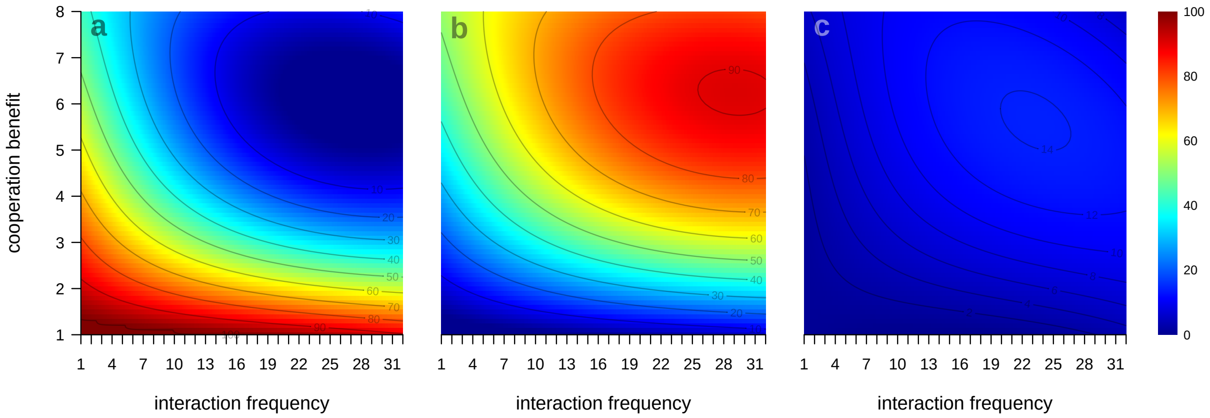

Supplementary Figure 19. Population composition across the parameter space. Estimated percentage of defectors (a), friend-focused agents (b), and incomplete Heider agents (c) as a function of the return on cooperation b and the interaction frequency i before adaptation takes place.

Summary

The above patterns can be summarised as follows:

(1) Heider agents and enemy-focused agents create large, densely interconnected communities.
(2) Agents that do not implement the 'enemy of my enemy is my friend' heuristic (i.e. friend-focused and incomplete Heider agents) create many small communities, instead.
(3) The 'enemy of my enemy is my friend' heuristic is vulnerable to invasions by defectors. Both, Heider agents and enemy-focused agents do not survive against defectors.
(4) Agents that do not implement the 'enemy of my enemy is my friend' (i.e. friend-focused and incomplete Heider agents) can survive against defectors but population-wide cooperation rates remain low, due to small community sizes.
(5) Incomplete Heider agents do not increase population-wide cooperation in a population of defectors, incomplete, and friend-focused agents.

Hence, the 'enemy of my enemy is my friend' heuristic is responsible for, both, the larger community size accompanied by increases in population-wide cooperation, as well as the invasions by defectors.

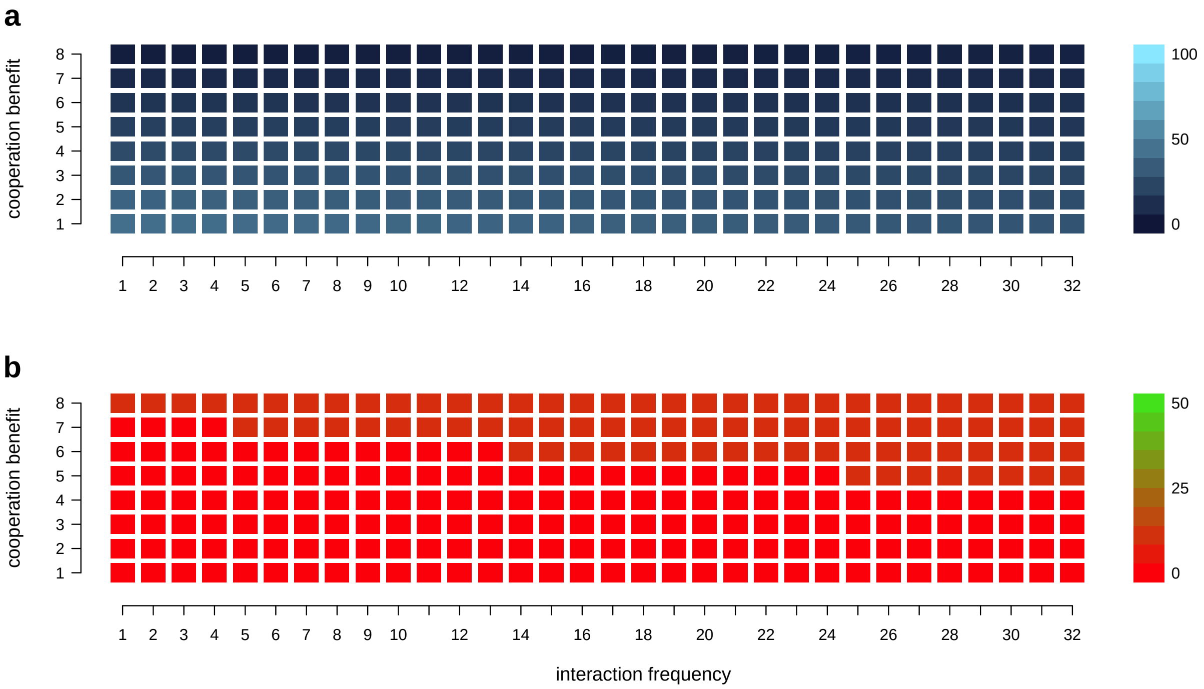

Supplementary Figure 20. Population characteristics across the parameter space. Panels show the relative proportion of incomplete Heider agents among incomplete Heider and friend-focused agents (a) and average frequency of mutual cooperation (b), depending on the return of cooperation (benefit b) and interaction frequency.

Supplementary Note 5

Changing the speed at which agents form relationships (r) does not alter the general observations of cycles between the three strategies, but impacts the speed of the fission-fusion dynamics and the observed frequency of each strategy across time (see Supplementary Figure 21 for examples). Across the parameter space, the speed of cycles, measured by the variance in agent composition across time increases with r, independent of the other parameters (Supplementary Figure 22, linear regression; β_r = 17.74). Further, the proportion of defectors decreases with more punishing relationship changes (linear regression; β_r = -44.70). Proportionally, the prevalence of friend-focused agents increases (linear regression; β_r = 34.61), independent of the other parameters in the model.

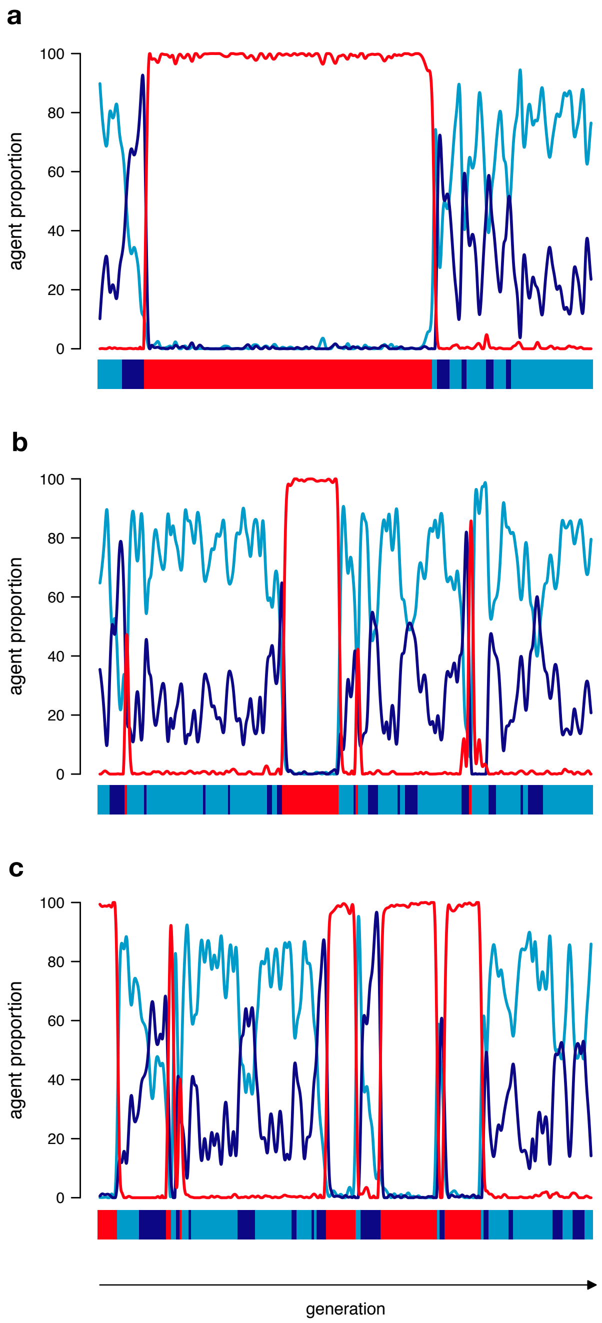

Supplementary Figure 21. Population cycles between a population that consists of friend-focused agents (light blue line), Heider agents (dark blue line), and free-riders (red line), across $r = 0.1$ (a), $r = 0.3$ (b), and $r = 0.5$ (c) – based on $n = 100$, 4×10^5 iterations, $i = 10$, $c = 1$, $b = 4$.

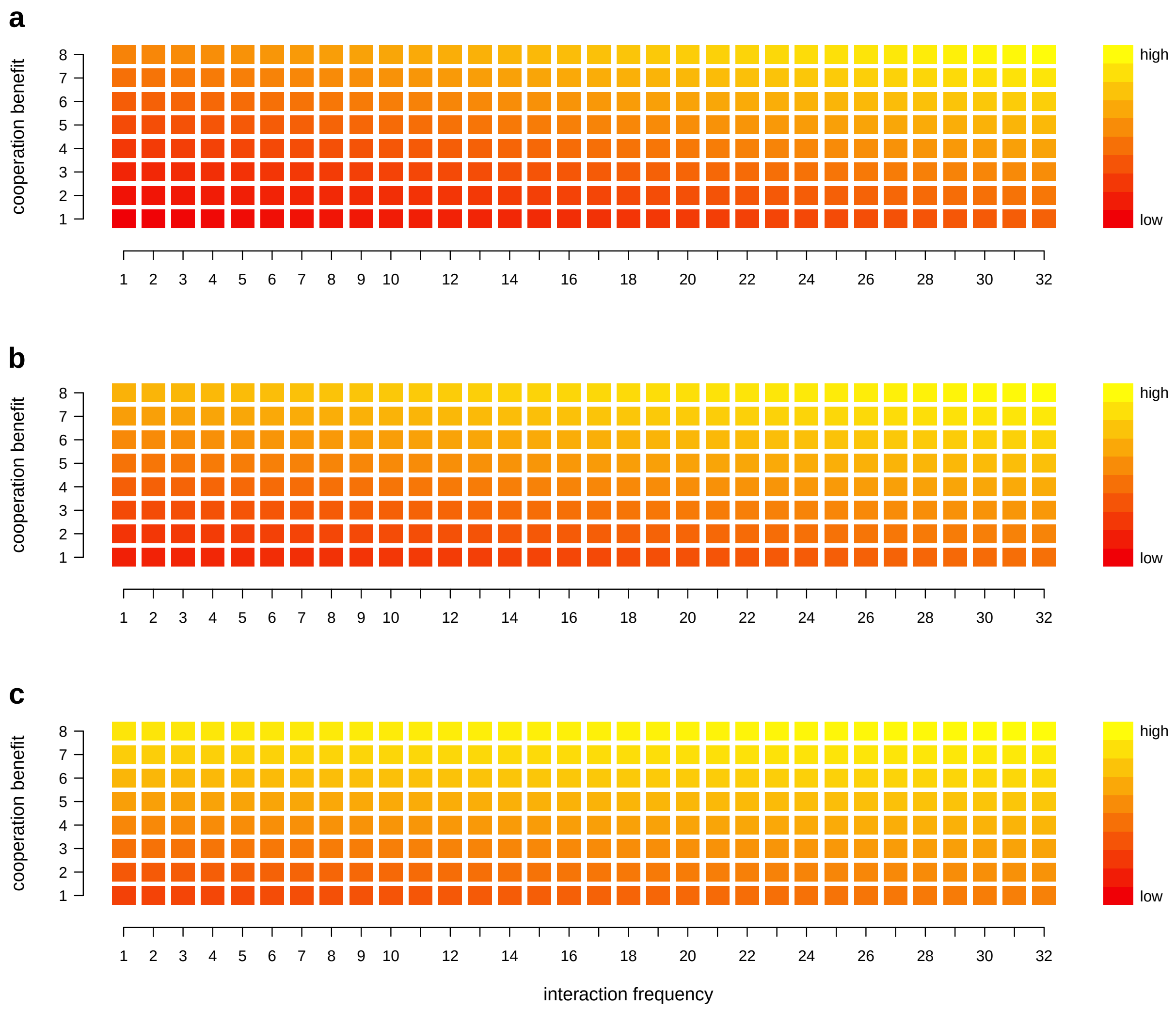

Supplementary Figure 22. Population instability, calculated by the variance in the agent-composition across time for $r = 0.1$ (a), $r = 0.3$ (b), and $r = 0.5$ (c), depending on the return of cooperation (benefit b) and interaction frequency.

Supplementary Note 6

So far, the evolutionary simulation results were based on a population of 100 agents. To see whether we observe the same patterns in larger populations, we repeated our simulations with Heider agents, friend-focused agents, and defectors in a population of $n = 500$ across the same parameter space. We further manipulated the speed of forming relationships r.

Supplementary Figure 23 shows the agent-composition across the parameter space (aggregated across mutation rate, as above). Similar to the simulations with $n = 100$, the proportion of defectors in the population decrease, while the proportion of Heider and friend-focused agents increase with increased benefit of cooperation and interaction frequency. The relative proportion of Heider agents remains low across the full parameter space.

As in simulations with $n = 100$, the proportion of defectors decreases with more punishing relationship changes. Aggregating across the parameter space, populations are comprised of 38% defectors, on average, with $r = 0.1$, dropping to 25% with $r = 0.3$ and $r = 0.5$. The relative proportion of friend-focused agents and Heider agents, consequently, increase from 48% with $r = 0.1$ to 59% with $r = 0.3$ and $r = 0.5$ for friend-focused agents, and from 14% with $r = 0.1$ to 16% with $r = 0.3$ and $r = 0.5$ for Heider agents.

Compared to smaller populations, larger populations allow reputation-based agents to survive already in an environment with intermediate return on cooperation and interaction frequency, since the relative fitness-advantage of single defectors is lower in larger populations. Supplementary Figure 24 exemplifies the population dynamic across time. As with a population of $n = 100$, we observe more frequent defector invasions the faster agents form relationships (r), keeping the other parameters constant.

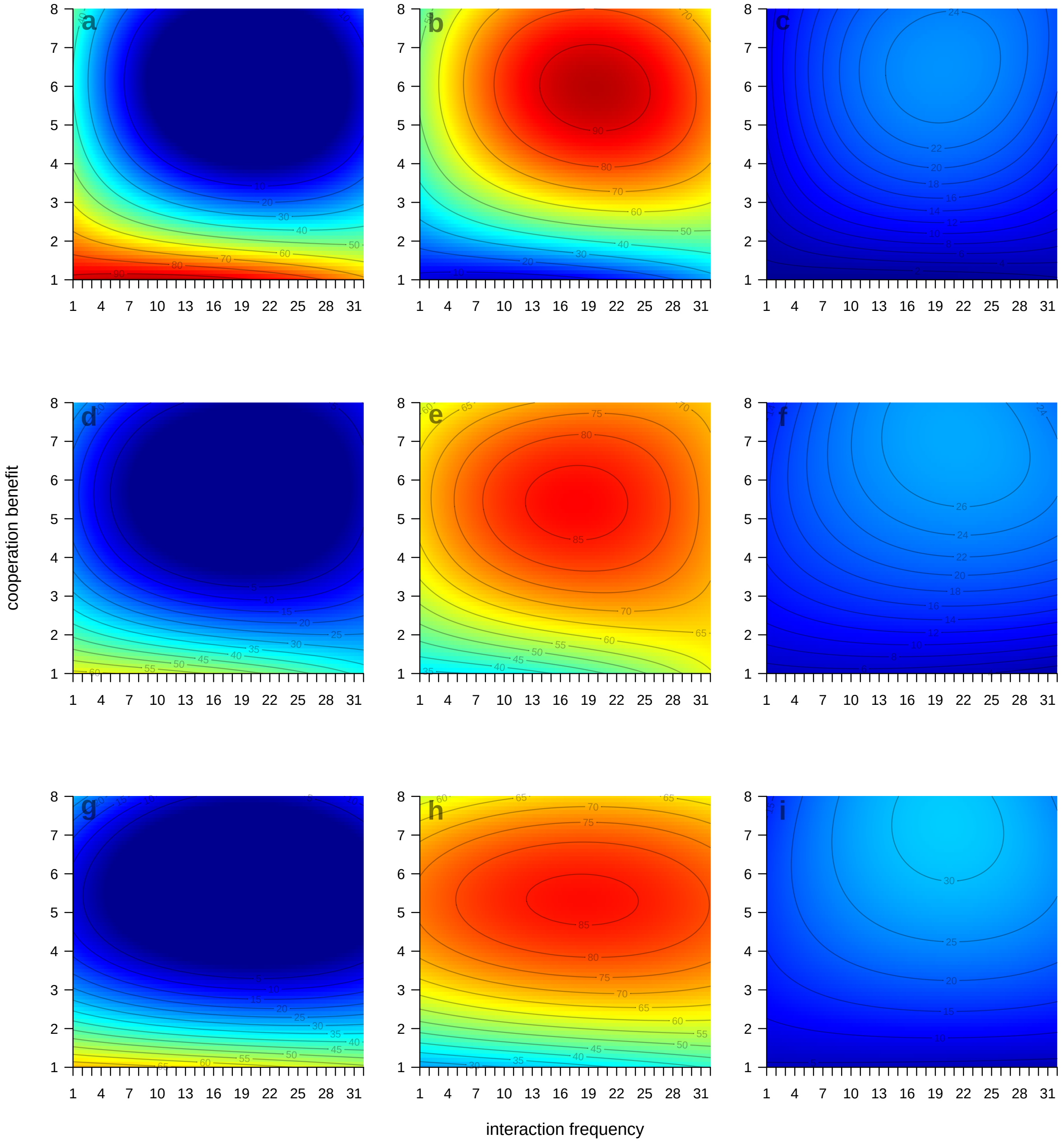

Supplementary Figure 23. Population composition across the parameter space. Estimated percentage of defectors (first column, a,d,g), friend-focused agents (second column, b,e,h), and Heider agents (third column, c,f,i), for slow ($r = 0.1$, first row, a,b,c), medium ($r = 0.3$, second row, d,e,f), and fast ($r = 0.5$, third row, g,h,i) relationship building, as a function of the return on cooperation b and the interaction frequency i before adaptation takes place.

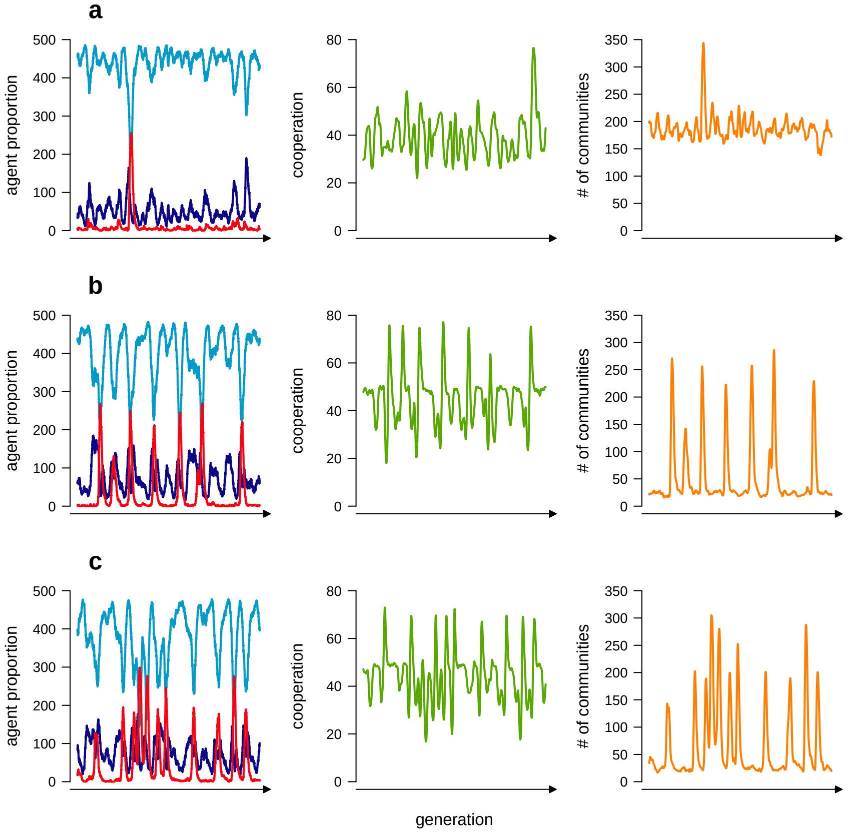

Supplementary Figure 24. Population composition (first column), cooperation (second column), and fission-fusion dynamic (third column) between a population that consists of friend-focused agents (light blue line), Heider agents (dark blue line) and free-riders (red line), across $r = 0.1$ (a), $r = 0.3$ (b), and $r = 0.5$ (c) – based on $n = 500$, 4×10^5 iterations, $i = 5$, $c = 1$, $b = 2$.

References

[1] Heider, F. Attitudes and cognitive organization. *The Journal of psychology* **21**, 107–112 (1946).

[2] Blondel, V. D., Guillaume, J.-L., Lambiotte, R. & Lefebvre, E. Fast unfolding of communities in large networks. *Journal of Statistical Mechanics: Theory and Experiment* **2008**, P10008–12 (2008).
