# Supplementary material for: The rise and fall of cooperation through reputation and group polarization
Source: Nat Commun. 2019 Feb 15;10:776. doi: 10.1038/s41467-019-08727-8 (PMC6377668; doi:10.1038/s41467-019-08727-8)
Supplement: Supplementary file 2 — Description of Additional Supplementary Files [file 41467_2019_8727_MOESM2_ESM.pdf]

Description of Additional Supplementary Files

File Name: Supplementary Movie 1
Description: Polarization. Example for the network development of n = 20 Heider agents. Each line indicates a positive relationship between two agents. Negative or zero relationships are omitted.

File Name: Supplementary Movie 2
Description: Small communities. Example for the network development of n = 20 friend-focused agents. Each line indicates a positive relationship between two agents. Negative or zero relationships are omitted

File Name: Supplementary Movie 3
Description: Community polarization. Example for the network development of n = 16 friend-focused agents (light blue circles) and n = 4 Heider agents (dark blue circles). Each line indicates a positive relationship between two agents. Negative or zero relationships are omitted.

File Name: Supplementary Movie 4
Description: Evolutionary dynamic. Example for the adaptation of strategies under selection pressure (n = 20, u = 0.01, i = 10, b = 4). Each line indicates a positive relationship between two agents. Negative or zero relationships are omitted. Agents change strategy according to the Moran process (change in colour, light blue circles = friend-focused agents, dark blue circles = Heider agents, red circles = defectors).
